# Supplementary figures and images for: Merging short and stranded long reads improves transcript assembly
Source: PLoS Comput Biol. 2023 Oct 26;19(10):e1011576. doi: 10.1371/journal.pcbi.1011576 (PMC10629667; doi:10.1371/journal.pcbi.1011576)

# S1 Fig

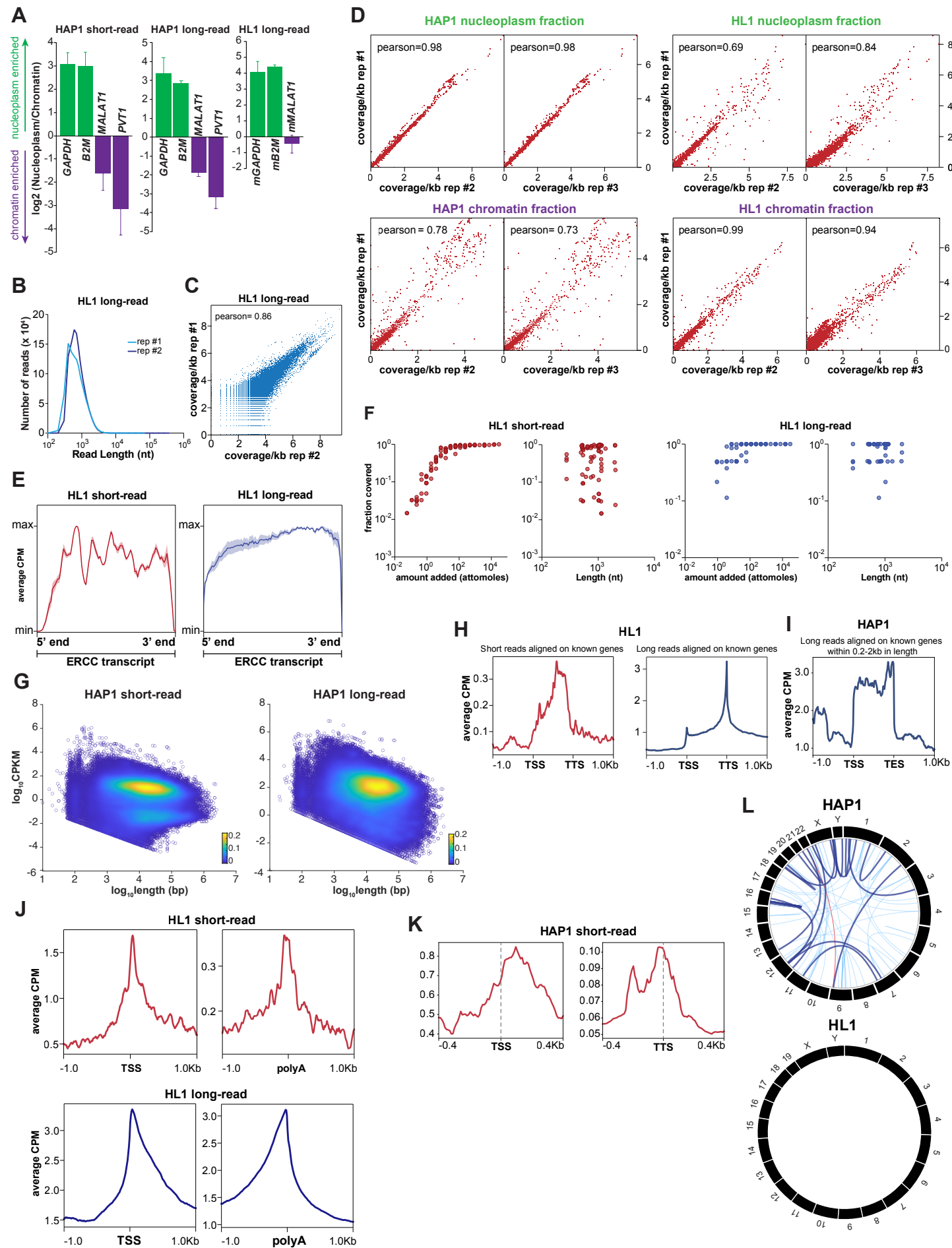

Supplement: S1 Fig — A. RT-qPCR confirmation of RNA fractionation into chromatin (purple) and nucleoplasm (green) extracts in the indicated samples. Protein coding transcripts (GAPDH and B2M) were used as representative of nucleoplasm enrichment while lncRNA (MALAT1 and PVT1) were used as controls for chromatin enrichment. Error bar indicates SD for replicates (n = 3 for short-read; n = 2 for long-read). B. Density plot of read lengths from long-read sequencing of HL1 replicates (n = 2). C. Correlation between HL1 replicates (n = 2) for genomic coverage of long-read alignments binned at 1 kb. Pearson correlation is shown. D. Correlation between HAP1 (left) and HL1 (right) replicates (n = 3) for genomic coverage of short-read alignments (top nucleoplasm fraction; bottom chromatin fraction) binned at 1 kb. Pearson correlations are shown for each plot. E. Average counts per million (average CPM, dark color; +/- SD, lighter color) of aligned reads across all ERCC transcripts, meta-scaled to 1000 nt, in the HL1 short-read (n = 3) and long-read (n = 2) samples. F. Relationship between the average fraction of ERCC transcripts covered by short (left) or long (right) read alignments as a function of their amount (attomoles) or length (nt) for HL1 samples. G. Relationship between length and CPKM (coverage per kb per million mapped reads) of known genes in HAP1 short- and long-read datasets. Average CPKM for replicates are plotted against length of genes on a log10 scale. Colors correspond to kernel density estimations of scatter plot distribution. H. Metagene plots of mapped read coverage (average CPM) for HL1 short- and long-read alignments, scaled to transcription start sites (TSS) and transcription termination sites (TTS) of known genes. I. Metagene plot of mapped read coverage (average CPM) for HAP1 long-read alignments, scaled to transcription start sites (TSS) and transcription termination sites (TTS) of a subset of known genes which are similar in length distribution (0.2–2 kb) to ERCC gen [file pcbi.1011576.s001.pdf]

**S2 Fig**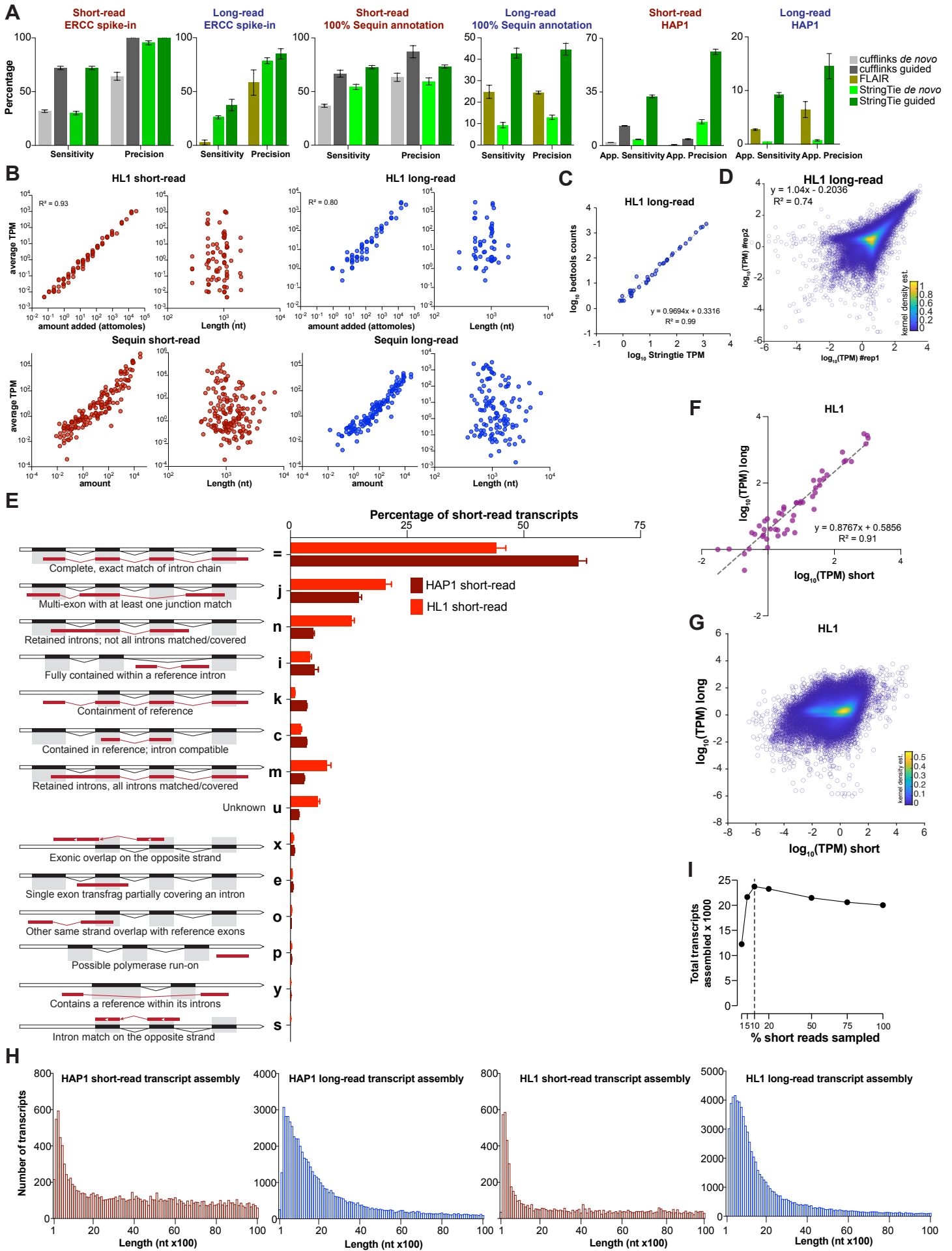

Supplement: S2 Fig — A. Comparison of the indicated short-read (left) and long-read (right) transcript assembly methods for ERCC, sequin and HAP1 transcripts. Shown are the sensitivity and precision of assembly at the transcript level as computed by gffcompare. Note that apparent (app.) sensitivity and precision indicate comparison to reference human annotations which does not reflect ground truth for a given cell line. Error bar indicates SD (n = 2–3). B. Top: Average abundance (Tags Per Million; TPM) of ERCC transcripts determined by StringTie versus amount added (attomoles) or length (nt) of the transcript in HL1 samples (n = 3 for short reads, n = 2 for long reads). Bottom: Average abundance (Tags Per Million; TPM) of sequin spike-in transcripts determined by StringTie versus amount added or length (nt) of the transcript in SGNex direct-cDNA samples of HCT116 samples (n = 3 for short and long reads). Short-read plots are shown in red (left), long-read plots in blue (right). R2, correlation coefficient for linear regression. C. Comparison of abundance (TPM) of ERCC transcripts obtained from StringTie with BEDTools counts for HL1 long-read replicate 1. Both methods performed nearly identically (R2 = 0.99, correlation coefficient for linear regression). D. Correlation between HL1 replicates for transcriptome assembly by StringTie. Shown are the scatter plots of abundance (TPM) of each transcript in the two replicates. Colors correspond to kernel density estimations of scatter plot distribution. R2, correlation coefficient for linear regression. E. Comparison of structure of transcripts assembled by StringTie for HAP1 and HL1 short-read samples with structure of reference genome transcripts. The class codes for relationship between the assembled transcript and the closest reference transcript were deduced from gffcompare. F. Correlation plot for the average abundance (TPM) of ERCC spike-in transcripts in the short-read datasets (n = 3) with the average abundance in the long-read dataset [file pcbi.1011576.s002.pdf]

**Figure S3**

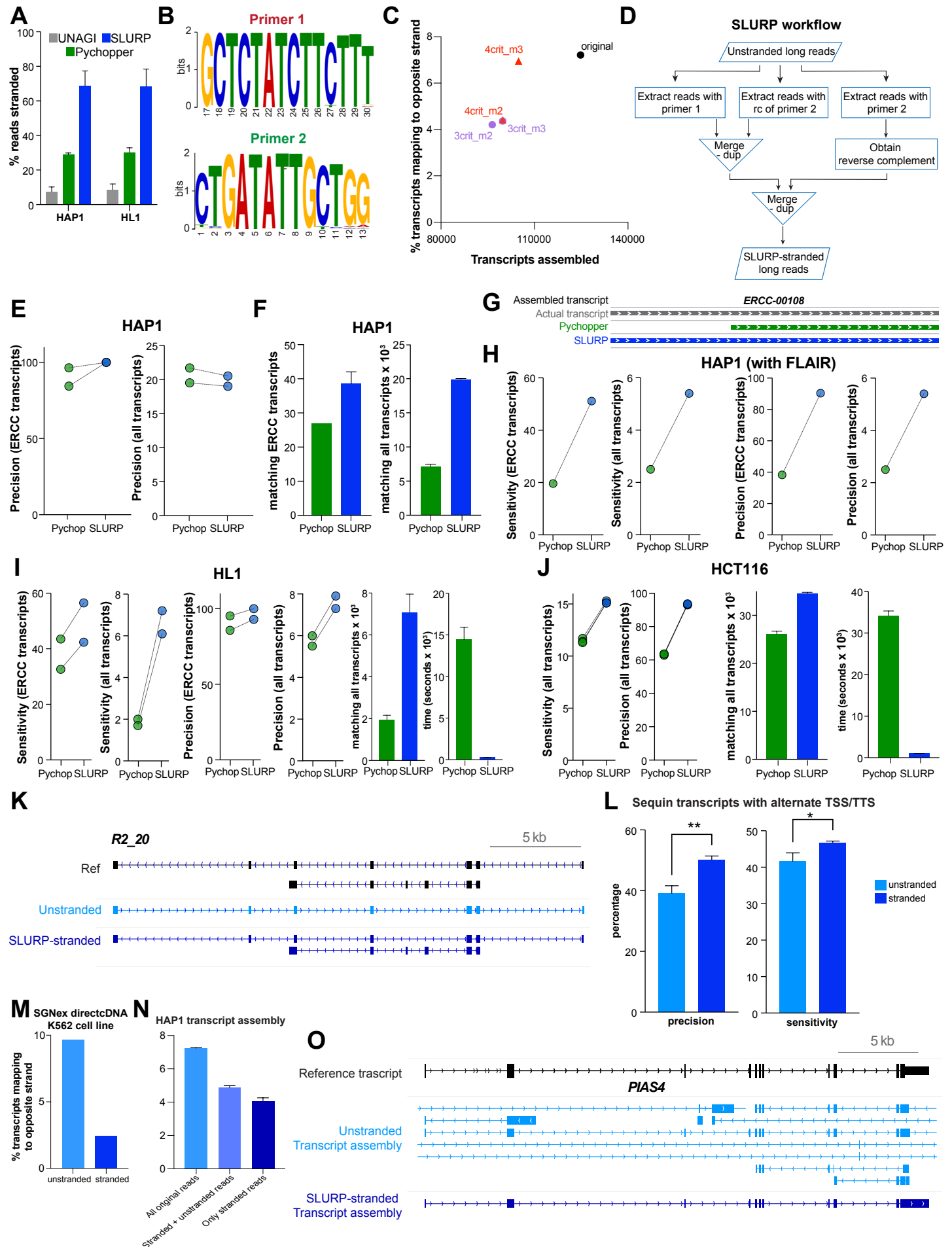

Supplement: S3 Fig — A. Percent of long reads stranded by UNAGI [65], Pychopper, and SLURP (Stranding Long Unstranded Reads using Primers) in the HAP1 and HL1 datasets. B. Detection of primer 1 and primer 2 sequences in the long reads using MEME [64] de novo motif discovery. C. Measurement of indicated stranding methods in terms of the number of transcripts assembled and extent of transcripts mapping to the wrong strand of the reference genome. 3crit indicates using primer 1, primer 2 and reverse complement of primer 2; 4crit indicates primer 1, reverse complement of primer 1, primer 2 and reverse complement of primer 2; m2 indicates allowing 2 mismatches and m3 indicates allowing 3 mismatches. D. Workflow of SLURP pipeline. Reads that contain the first-strand synthesis primer (primer 1) or reverse complement (rc) of the strand switching primer (primer 2) are non-redundantly merged with the reverse complement of reads that contain the strand switching primer. E. Comparison of Pychopper and SLURP for the precision of assembling the ERCC (left) or all (right) transcripts in the HAP1 dataset. F. Comparison of Pychopper and SLURP for the total number of matching ERCC (left) or all (right) transcripts in the HAP1 dataset. G. Example of an ERCC transcript correctly assembled using SLURP where Pychopper failed to do so. H. Comparison of Pychopper and SLURP for the sensitivity and precision of transcript assembly by FLAIR in the HAP1 dataset. I. Comparison of Pychopper and SLURP for the sensitivity and precision of transcript assembly, total number of matching transcripts, and the computational time required for the stranding of reads in the HL1 dataset. J. Same as I, for the HCT116 dataset from the SG-Nex consortium. K. Example of a sequin spike-in transcript annotation with two spliced isoforms that vary in TSS and TTS, that is correctly assembled by SLURP-stranded long reads where the original unstranded reads failed to do so. L. Comparison of SLURP-mediated change in the sensitivity and pre [file pcbi.1011576.s003.pdf]

S4 Fig

A

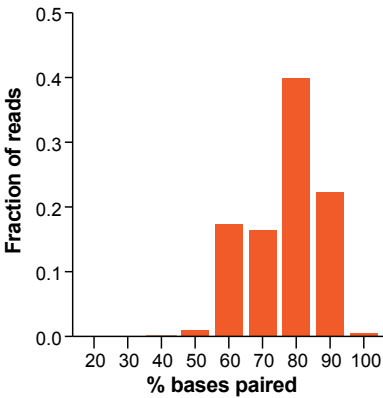

Supplement: S4 Fig — A. Bar plot of percentage of paired bases in the artifactual reads as a test for palindromes in the reads. RNAfold was used to determine paired bases in the secondary structure where a palindrome outweighs other structures. (PDF) [file pcbi.1011576.s004.pdf]

S5 Fig

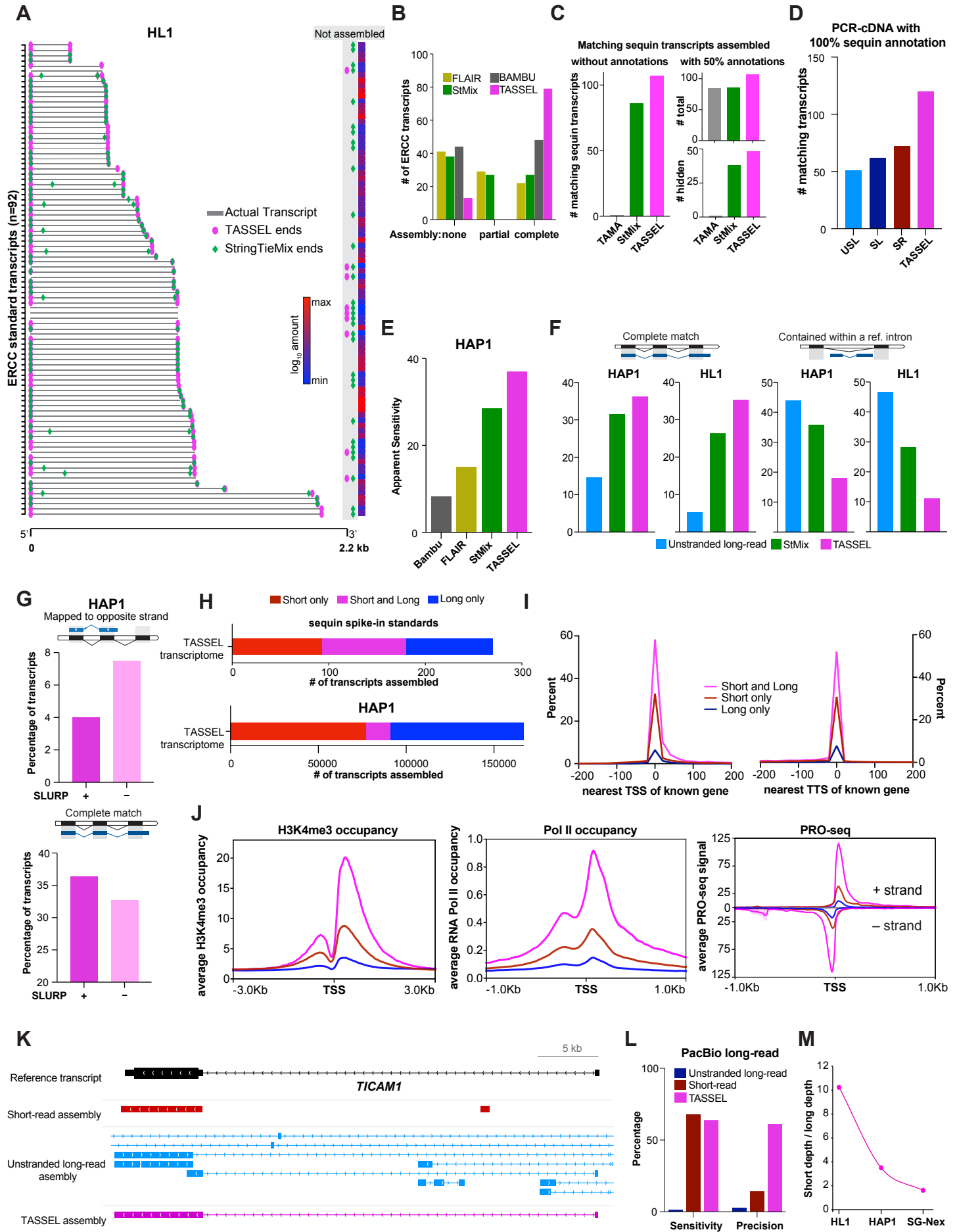

Supplement: S5 Fig — A. Ends of the 92 ERCC transcripts (arranged in increasing order of length) assembled by TASSEL (magenta circle) and StringTie Mix (StMix, green diamond) in the HL1 dataset. Gray bar indicates actual transcript. The color bar indicates the abundance of the given transcript. B. Number and extent of ERCC standard transcripts assembled by the indicated assembly method in the HAP1 dataset. StMix: StringTie Mix. SLURP-stranded reads were used for FLAIR assembly. C. Number of matching sequin transcripts assembled by the indicated method when no reference sequin annotation (left) or 50% of the reference annotations (middle and right) were provided to TAMA at the merge stage. Note that no sequin annotation was provided to StringTie Mix or TASSEL at the merge stage for any of the comparison points here. D. Number of total matching sequin transcripts assembled by the indicated assembly method with ONT PCR-cDNA sequencing kit. E. Sensitivity of the indicated assembly methods at the locus level for the HAP1 dataset. Transcriptome assembled by the given method in the HAP1 dataset was compared against reference annotation (gencode hg38v35) using gffcompare. SLURP-stranded reads were used for FLAIR assembly. Apparent sensitivity indicates use of gencode annotation as ground truth. F. Percent of assembled transcripts that match completely with a transcript (left) or are contained within an intron (right) of the reference transcript, using only long-read assembly, StringTie Mix or TASSEL in the HAP1 and HL1 datasets. G. Comparison of inclusion or omission of SLURP stranding in TASSEL. Shown are percentage of transcripts mapped to the opposite strand (top) or completely matching (bottom) the reference transcripts in the HAP1 dataset. H. Number of transcripts contributed by short- and long-read assemblies to the TASSEL transcriptome in the sequin and HAP1 datasets. Long or short-read transcripts were used as a query against the TASSEL merged transcriptome as a reference in gffcompare. [file pcbi.1011576.s005.pdf]

S6 Fig

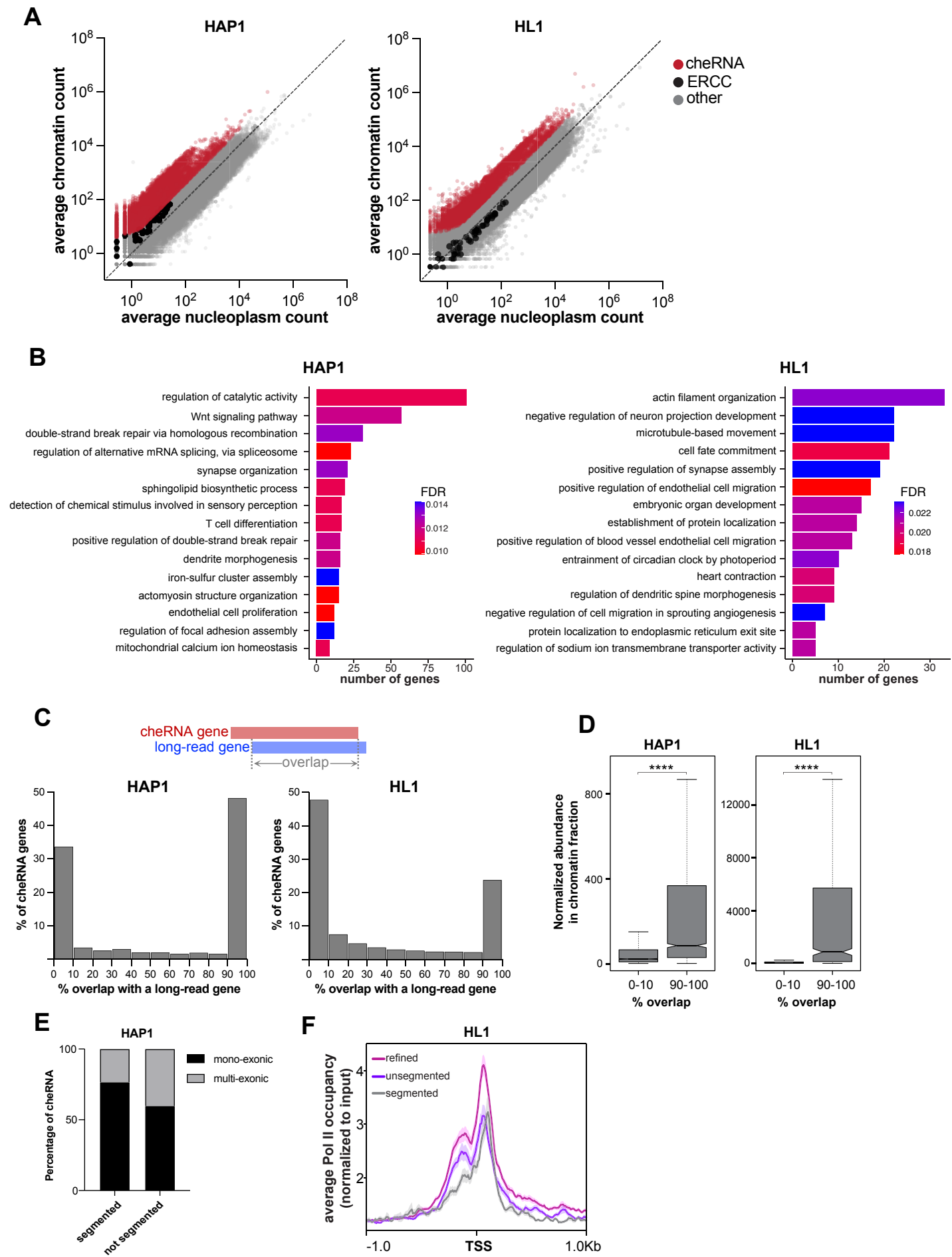

Supplement: S6 Fig — A. Average count of genes in the chromatin and nucleoplasm fractions in HAP1 (left) and HL1 (right). Normalized gene counts obtained through DESeq2 were averaged for replicates (n = 3). B. Gene ontology enrichment analyses of protein-coding genes closest to cheRNA genes detected in HAP1 and HL1 datasets. Top 15 categories detected by DAVID [101] and associated number of genes are shown. Color bars correspond to false discovery rate (FDR). C. Histogram depicting the extent of overlap between cheRNA genes from short-read assembly and genes assembled from long-read assembly in HAP1 and HL1 samples. D. Comparison of the abundance (normalized DESeq2 gene count) of cheRNA genes showing minimum (0–10%) and maximum (90–100%) overlap with a corresponding long-read gene. Data points outside 1.5x of Inter-quartile range were removed as outliers. **** p < 0.0001, Two-tailed Mann-Whitney test. E. Prevalence of mono and multi-exonic cheRNA genes in segmented or unsegmented cheRNA genes. F. Metagene plot depicting average RNA Pol II occupancy (solid lines; shaded regions indicate SE) at the TSS (±1Kb) of segmented, unsegmented and TASSEL-refined cheRNA genes in the HL1 dataset. RNA Pol II occupancy was calculated from ChIP-seq data from heart of an eight-week-old mouse [102]. (PDF) [file pcbi.1011576.s006.pdf]
