# Supplementary material for: Merging short and stranded long reads improves transcript assembly
Source: PLoS Comput Biol. 2023 Oct 26;19(10):e1011576. doi: 10.1371/journal.pcbi.1011576 (PMC10629667; doi:10.1371/journal.pcbi.1011576)
Supplement: S1 Note — (DOCX) [file pcbi.1011576.s007.docx]

**S1 Note: Long-read alignments capture novel cell line-specific gene fusion events**

An important feature that can be detected with long reads is gene fusion. Long reads are particularly useful in the reliable detection of gene fusions, as they not only detect the junction but also are more likely to cover uniquely mappable sequences on both sides of the discontinuity. Using LongGF [1] with long reads from HAP1 cells, we detected >20 gene fusion events supported by at least two reads in each of the two HAP1 long-read replicates (S1L Fig). Notably, we were able to detect the BCR::ABL fusion which is a key mutagenic fusion in this leukemia cell line [2]. We compared gene fusion events in our long-read transcriptome data with a comparable long-read genomic DNA dataset in HAP1 cells [3]. Surprisingly, we found only one common fusion, which was BCR::ABL. Therefore, our long-read transcriptome analyses unveiled novel gene fusion events in the HAP1 cells, possibly due to enhancement of sensitivity in our chromatin-focused pool of transcripts. Intriguingly, we did not detect any gene-fusion in the non-leukemia HL1 cells (S1L Fig), suggesting a cell line-specific detection of translocations in our dataset that is not due to technical artifacts in the library preparation.

**References**

1. Liu Q, Hu Y, Stucky A, Fang L, Zhong JF, Wang K. LongGF: computational algorithm and software tool for fast and accurate detection of gene fusions by long-read transcriptome sequencing. BMC genomics. 2020;21(Suppl 11):793. Epub 2020/12/30. doi: 10.1186/s12864-020-07207-4. PubMed PMID: 33372596; PubMed Central PMCID: PMCPMC7771079.

2. Andersson BS, Collins VP, Kurzrock R, Larkin DW, Childs C, Ost A, et al. KBM-7, a human myeloid leukemia cell line with double Philadelphia chromosomes lacking normal c-ABL and BCR transcripts. Leukemia. 1995;9(12):2100-8. Epub 1995/12/01. PubMed PMID: 8609723.

3. Law WD, Warren RL, McCallion AS. Establishment of an eHAP1 human haploid cell line hybrid reference genome assembled from short and long reads. Genomics. 2020;112(3):2379-84. Epub 2020/01/22. doi: 10.1016/j.ygeno.2020.01.009. PubMed PMID: 31962144.
