# Supplementary material for: Merging short and stranded long reads improves transcript assembly
Source: PLoS Comput Biol. 2023 Oct 26;19(10):e1011576. doi: 10.1371/journal.pcbi.1011576 (PMC10629667; doi:10.1371/journal.pcbi.1011576)
Supplement: S1 Table — (PDF) [file pcbi.1011576.s008.pdf]

Sample library	Reads generated	Reads passing QC	Reads mapped	Bases mapped	Median read length
HAP1 long-read chromatin fraction #1	1.24 million	1.13 million	96.84%	90.83%	1.97kb
HAP1 long-read chromatin fraction #2	2.94 million	1.82 million	99.03%	90.06%	1.7kb
HL1 long-read chromatin fraction #1	1.79 million	1.55 million	93.50%	86.24%	1.39kb
HL1 long-read chromatin fraction #2	2.5 million	1.68 million	96.51%	85.26%	1.26kb
HAP1 short-read chromatin fraction #1	127.27 million	127.27 million	96.67%	96.43%	51 nt
HAP1 short-read chromatin fraction #2	127.82 million	127.82 million	96.44%	96.20%	51 nt
HAP1 short-read chromatin fraction #3	129.68 million	129.68 million	96.54%	96.29%	51 nt
HAP1 short-read nucleoplasm fraction #1	126.03 million	126.03 million	97.64%	97.36%	51 nt
HAP1 short-read nucleoplasm fraction #2	126.85 million	126.85 million	97.70%	97.42%	51 nt
HAP1 short-read nucleoplasm fraction #3	131.55 million	131.55 million	97.73%	97.46%	51 nt
HL1 short-read chromatin fraction #1	354.05 million	354.05 million	87.10%	86.81%	51 nt
HL1 short-read chromatin fraction #2	337.90 million	337.90 million	83.88%	83.56%	51 nt
HL1 short-read chromatin fraction #3	327.32 million	327.32 million	85.37%	85.05%	51 nt
HL1 short-read nucleoplasm fraction #1	335.65 million	335.65 million	77.07%	77.65%	51 nt
HL1 short-read nucleoplasm fraction #2	327.32 million	327.32 million	65.43%	65.02%	51 nt
HL1 short-read nucleoplasm fraction #3	349.83 million	349.83 million	72.96%	72.61%	51 nt

S1 Table: Sequencing output of short- and long-read RNA-seq.
