# Supplementary material for: Merging short and stranded long reads improves transcript assembly
Source: PLoS Comput Biol. 2023 Oct 26;19(10):e1011576. doi: 10.1371/journal.pcbi.1011576 (PMC10629667; doi:10.1371/journal.pcbi.1011576)
Supplement: S2 Table — (PDF) [file pcbi.1011576.s009.pdf]

| Primer name | Sequence |
|---|---|
| human_GAPDH F | CCGGGAAACTGTGGCGTGATGG |
| human_GAPDH R | AGGTGGAGGAGTGGGTGTCGCTGTT |
| human_B2M F | CTCTCTCTTTCTGGCCTGGAG |
| human_B2M R | TCTGCTGGATGACGTGAGTA |
| human_PVT1 F | CTGAGCCCCACTTCCTCTTG |
| human_PVT1 R | TCATCACGCTCCCCTAGCTT |
| human_MALAT1 F | GGAGCTTGAGGAAACCGCAGATAAG |
| human_MALAT1 R | GCTTCATCTCAACCTCCGTCATG |
| mouse_GAPDH F | CATCACTGCCACCCAGAAGACTG |
| mouse_GAPDH R | ATGCCAGTGAGCTTCCCGTTCAG |
| mouse_B2M F | CATGGCTCGCTCGGTGAC |
| mouse_B2M R | CAGTTCAGTATGTTCGGCTTCC |
| mouse_MALAT1 F | CGTTTGAAGGCATGAGTTGG |
| mouse_MALAT1 R | TGCCTCCCAAGTGCTAGGAT |

**S2 Table: Primers used for RT-qPCR analyses.**
