# Supplementary material for: Merging short and stranded long reads improves transcript assembly
Source: PLoS Comput Biol. 2023 Oct 26;19(10):e1011576. doi: 10.1371/journal.pcbi.1011576 (PMC10629667; doi:10.1371/journal.pcbi.1011576)
Supplement: Attachment — Submitted filename: Kainth_Haddad_ResponseLetter.pdf [file pcbi.1011576.s010.pdf]

We would like to sincerely thank the reviewers for their thoughtful critiques, excellent experimental suggestions, and time invested in critically evaluating our manuscript. We have taken their suggestions to heart and believe that the critiques have helped guide our revisions to markedly improve the paper, especially the consensus on using alternatively spliced spike-in standards to benchmark our pipelines. Below, we respond to each point raised by the reviewers, and indicate how we address their concerns, often with additional analyses and data added to the manuscript. The line numbers in this rebuttal letter correspond to those in the main manuscript.

The major concerns of the reviewers, which will need to be sufficiently addressed in a revision, center around the evaluation of the transcriptome assemblies. In particular, there were concerns that (1) the precision of TASSEL is not appropriately evaluated (2) the straightforward use of a reference annotation as a ground truth is misleading and (3) use of ERCC RNA standards only allows for the evaluation of assembly performance for monoexonic transcripts. I have a few comments in addition to those of the reviewers that I hope you would also address in a revision:

1. Given the emphasis of PLoS Computational Biology on computational methods, it would be helpful to have additional experiments that explore the individual contributions of the novel components of TASSEL to its performance. The two main components appear to be (A) the stranding of long reads with SLURP and (B) the strand-aware merge of the long and short-read assemblies. Computational experiments that leave out one or both of these components could be performed to determine how performance is affected.

For example, how does TASSEL perform without the SLURP stranding?
This is a reasonable suggestion as SLURP was presented as an integral component of TASSEL. We have now explicitly tested this point-- the new Fig S5E shows reduced efficacy of TASSEL without SLURP in terms of mapping to the correct strand as well as complete match with the reference transcripts.

Or, how well does TASSEL perform with only the short-read data, or only the long-read data?
To address this, we directly compared TASSEL with only its short-read or long-read components. As shown in the new Figs 5F and 5G, TASSEL performs better than either of the datasets alone in terms of sensitivity of detection as well as precision at higher stringency levels. Consensus transcripts in TASSEL are also better indicators of transcription start and termination sites (new Figs S5F, S5G and S5H) than the transcripts derived from either short- or long-read assembly alone.

If you provide SLURP stranded long-reads to the other assemblers, does that also improve their performance?
This is a great suggestion as it could expand the applicability of SLURP. In the revised manuscript, we show that stranding via SLURP increases the efficacy of another long-read assembler, FLAIR (new Fig 3F and 3G). Furthermore, we only tested SLURP for the ONT direct-cDNA kit in the original manuscript. Expanding the scope of SLURP, we now show that SLURP stranding can be used for the ONT PCR-cDNA kit as well (new Fig 3F and 3G).

2. Related to the reviewers' concerns regarding the evaluation, I am concerned about the validity of using a reference annotation as a comparison for the output of "guided" assemblers, which use that

same reference annotation as an input (i.e., the results in Figure S2A). Perhaps there are alternative evaluation methods that could be used.
We think the reviewer's request to use a better reference annotation to evaluate the assembly programs is a great idea. We addressed this concern in multiple ways. First, we used existing short- and long-read datasets from the SG-Nex consortium with sequin spike-in standards to re-do all of our evaluations. Based on the common concerns from all three reviewers regarding the lack of splicing and isoform variants in our original ERCC-based evaluations, we reasoned that inclusion of multi-exonic sequin spike-in standards would alleviate their concerns (new Figs S2A, 3F, 3G, 5E and S5B). Second, in separate analyses, we provided 50% of sequin annotations to assembly programs and tested how well they can assemble total as well as "hidden" transcripts (assembly withheld) to test guided and *de novo* assembly capabilities (new Figs 5F and 5G). Finally, at the places where we used mammalian reference annotation as the "ground truth", we explicitly cautioned the readers of the underlying caveat of such comparison (lines 246-249, 367-370, S2A Fig and S5C Fig). However, we do feel that it is important to test assembly methods on mammalian transcripts as they are not only more complex than any spike-in controls but also provide "real-world" information about the efficacy of these tools.

3. Figure 2F - should the x-axis label be "% of short-read transcripts" or "% of long-read transcripts"? The text corresponding to this figure is contradictory (lines 261-265).
Thanks for pointing out this discrepancy. The x-axis label should be "% of short-read transcripts". We have revised the corresponding text (lines 289-293).

--**Reviewer #1**--

Reviewer #1: In this paper, Kainth and Haddad et al create TASSEL, a pipeline that combines short and long read RNA seq data for transcriptome assembly. They highlight the unique advantages of each method and by leveraging each of their strengths they claim they can improve the sensitivity and accuracy of transcript assembly. TASSEL is based on the preexisting transcript assembly tool Stringtie2 which was used for assembling both data types and merging the transcriptomes. In the process of creating this pipeline they identified and subsequently removed a prevalent direct cDNA artifact. They also develop SLURP, which attempts to strand direct cDNA reads by using the Nanopore sequencing adapters that are theoretically present at the end of each read. To benchmark their pipeline they tested it on cheRNA data that they produced and validated their transcript assemblies using external TSS and TES databases and by performing H3K4me3, RNA Pol II enrichment, and PRO-seq. This work includes an in depth comparison between short and long-read technologies, as well as comparisons to contemporary long-read transcript assembly tools. However I do believe there are a few aspects that are missing in the evaluation of TASSEL in order for it to be claimed to be an improvement over other tools. Many of the figures, and legends, and methods can be improved in order to communicate exactly what was done and allow for reproducibility. I have included my major and minor comments below which I believe will help make the claims in this manuscript more robust.

Major comments

R1.1 Evaluation
Crucially, it is claimed that TASSEL uses the advantages of both types of RNAseq data, however it is also possible that it combines the disadvantages of both data too. One example is the challenge of

performing transcript assembly using short-reads, which can lead to the construction or combination of exons that do not exist. This is especially problematic for long multi-exon transcripts and can result in reporting transcripts that are not present in the sample. The rate of false positives is however not measured in TASSEL and it is possible that by combining these two datasets, the precision of the output is worse than other tools.

We thank the reviewer for raising this important concern. Since sensitivity (rate of false negative) and precision (rate of false positive) typically follow an inverse relation in most assembly methods, we compared these two parameters of TASSEL with other assemblers. While the baseline precision of TASSEL was lower than the StringTie Mix assembly, we show that equivalent levels of precision can be achieved with TASSEL by increasing the stringency of transcript detection as a function of minimum read counts required for transcript assembly (new Fig 5G). Importantly, even at the higher stringency levels, TASSEL maintains higher sensitivity of assembly than other assembly methods. In comparison, the sensitivity of StringTie Mix is not only lower than TASSEL at the baseline stringency but also declines precipitously at higher stringency. So, TASSEL provides much higher sensitivity than other methods with an option to increase precision without sacrificing sensitivity. The users can choose the stringency levels as per their requirement. These attributes are now detailed in the corresponding results (lines 430-435) as well as in the discussion section (lines 649-652).

There are additional ways TASSEL should be evaluated:
R1.1a The precision of TASSEL needs to be evaluated on a complex transcriptome and not only the spike-ins. ERCC spike-ins are single-exon by design and therefore not a challenge for short-reads to construct. The writers should measure the precision of their merged output, this can be done in a few ways. Using more complex multi-exon spike ins, simulated data so that the underlying ground truth is known or remove a sample of annotations to act as a test set and attempt to identify them.
We agree with the reviewer that the ERCC spike-in standards may not reflect the complexity of spliced transcripts typically found in the real datasets. We highlighted this caveat at lines 217-220 of the revised manuscript. To overcome this caveat, we tested SLURP and TASSEL on the existing short- and long-read datasets from the SG-Nex consortium for complex multi-exonic sequin spike-in standards. We report that SLURP not only corrected the strand of transcript assembly but also improved the overall sensitivity and precision of the sequin transcriptome (new Figs 3F and 3G). We also evaluated TASSEL on the sequin transcriptome. When provided with 100% of sequin annotations, TASSEL showed the highest sensitivity compared to other methods (new Fig 5E). Per this reviewer's suggestion, we also tested TASSEL by randomly removing 50% of sequin transcripts from the annotations prior to assembly. Here again, TASSEL assembled the highest number of correct "total" as well as "hidden" transcripts (new Fig 5F).

Additionally, although the gencode annotations do not provide the exact ground truth for a given cell line, we feel that it is important to test assembly methods on mammalian transcripts as they are not only more complex than any spike-in controls but also provide "real-world" information about the efficacy of these tools. In that line, we would like to re-iterate our findings from the comparison of TASSEL with StringTie Mix to detect transcription start and termination sites in the human transcriptome in our HAP1 dataset. We used multiple molecular features (H3K4me3, Pol II occupancy and PRO-seq) defined in the HAP1 cell line to probe transcript ends. While not ground truth, they are

derived from experimental data in the same cell line, and therefore are more tailored to the HAP1 transcriptome than the gencode assembly derived from a host of different cell types. All the derived analyses (Figs 5I and 5J), effectively done on complex mono- as well as multi-exonic transcripts, show that TASSEL performs better than StringTie Mix. As alternative TSS and TTS are the major driver of isoform variation across human tissues (Reyes et al., *Nucleic Acids Research*, 2018) – even more so than internal splicing – we feel that improvement in detection of TSS and TTS by TASSEL, irrespective of internal exon structure, is a significant contribution to the field. For these reasons, we retained many of the original analyses done on ERCC as well as cell-line specific transcriptomes.

We feel that these multiple lines of evidence make a compelling case that TASSEL provides improvement in discovery as well as assembly of mono- and multi-exonic transcripts.

R1.1b TASSEL should not only be compared to long-read assembly tools but also tools that only do assembly using the short-read data. This should highlight the expected difference between the long-read and short-read exclusive methods and hopefully will show that TASSEL is the union of positive differences.

As suggested by the reviewer, we compared TASSEL with only short-read and only long-read transcript assembly in terms of sensitivity and precision of sequin spike in assembly by providing 50% of the sequin transcriptome annotations. First, TASSEL assembled the highest number of "total" as well as "hidden" transcripts than either of the short- or long-read assemblies alone (new Fig 5F). Second, TASSEL showed higher sensitivity and precision of transcript assembly than long-read assembly alone across a spectrum of stringency conditions. Although the baseline precision of TASSEL was lower than only the short-read assembly, we show that TASSEL's precision can be substantially enhanced by increasing the stringency of transcript detection on the basis of the minimum number of read counts required to call a transcript. This limitation of TASSEL as well as the method to overcome the limitation is detailed in the manuscript (lines 430-435 and 649-652) so that the users can choose stringency levels as per their requirement.

R1.1c Many of the comparisons and evaluations of TASSEL are measured in percentage, however because of coverage differences it is likely the absolute number of transcripts detected is very different between the short-read and long-read data. It would be good to know how many transcripts in the final merged assembly are derived from the short-read, the long-read or both as without these numbers it is possible one data source is dominating the contribution to the final merged output. The subsequent TSS and TES analysis should then be separated between transcript models that were detected solely from each method and from both to show the contribution of each of these tools to detecting accurate transcript ends.

This is a fair point and we have now performed detailed analyses of the contribution of each of the individual components of TASSEL. In the new Fig S5F, we report that the short- and long-read platforms contribute equivalent numbers of matching transcripts to the TASSEL transcriptome. This is perhaps due to the reason that equal weightage is given to transcripts from both platforms during the TASSEL merge. Furthermore, we show that the consensus transcripts in the TASSEL transcriptome evince better enrichment of TSS and TTS (new S5G Fig) as well as H3K4me3 occupancy, Pol II occupancy and PRO-seq signal (new S5H Fig) than the transcripts obtained from either of the short- or long-read transcriptomes alone. Furthermore, as per the reviewer's suggestion, we have now included

an example where TASSEL correctly assembles a known spliced human transcript where the original short- or long-read dataset failed to do so (new S5I Fig).

In conjugation with point R1.1b, these analyses support the idea that TASSEL-mediated assembly leads to improvement in transcript assembly compared to short- or long-read assembly alone.

R1.2 Direct cDNA is discontinued
Two of the major advancements in TASSEL is the identification of a reverse transcription artifact and stranding of direct cDNA data. With the announcement that ONT is discontinuing the direct cDNA sequencing library preparation, can the authors comment on if their approach is applicable in other forms, such as to PacBio or PCR cDNA ONT sequencing.
We note that direct cDNA kits were discontinued 6 months after our submission of this manuscript to PLoS Computational Biology, but the present available technology does render this suggestion to test SLURP and TASSEL on other library preparation methods even more important. We now show that, in addition to the direct cDNA kit, SLURP and TASSEL can also be effectively used on currently available ONT PCR-cDNA kit data (new Figs 3F, 3G, S5B) as evidence of their applicability with contemporary long-read library preparation methods. Additionally, although the direct cDNA kit is discontinued, there is still a large amount of existing direct cDNA data where SLURP and TASSEL can be used to correct transcript assembly.

As for the PacBio sequencing, we found that the inherent lima correction of its Iso-seq platform orients the reads in the correct direction. Therefore, we think that SLURP may not provide extra benefit to strand PacBio long reads. However, as we observed for TASSEL with ONT reads, our proof of principle test shows that the merge of stranded PacBio reads with a corresponding short-read dataset improves transcript assembly (new S5J Fig). These aspects are now added in the discussion section (lines 670-675) of the revised manuscript.

R1.3 Treatment of reads that are still unstranded after processing
By stranding the direct cDNA reads, the authors claim to reduce the occurrence of mirrored transcripts being called on both strands. It is not clear however what is done with reads that are unable to be stranded due to lacking unambiguous barcodes. Including these reads as stranded can incur false confidence in downstream applications. The authors note that "Transcripts that are still assembled on the opposite strand could be a result of a residual pool of reads that were not stranded" however it is not clear why they would keep these reads in the analysis. They should repeat the analysis by removing the reads that were unable to be stranded to confirm this hypothesis, and see if this leads to a better transcript assembly.
We apologize for the ambiguity in the original phrasing. We do remove the reads that were unable to be stranded. We have re-written the corresponding texts in the result section (lines 370-373). In fact, we have included a new analysis to show that retention of unstranded reads reduces the efficacy of SLURP (new S3G Fig).

Minor comments:
R1.a In the abstract line 23 it is stated that long-read transcript assembly lacks strand of origin, but this is not entirely true because dRNA is stranded, and there are library preparations that allow stranded sequencing.

We have now revised the statement to explicitly mention cDNA-based methods.

R1.b Figure 1h is unclear and could be explained better in the main text and legend. Figure D shows that at the ends of the transcript there is only a one directional drop before/after the TSS/TES, however figure 1h suggests there is a drop at both ends. Is this just showing the coverage of the read ends? If so this should be made clear. Furthermore the legend says there are heatmaps but there are none present.
The apparent discrepancy arises due to the scaling of the two coverage plots. For Fig 1D, the coverage of short-read alignment is plotted across “average ERCC gene” meta scaled to 1kb. As the 5’ and 3’ end of the ERCC transcripts also demarcate the ends of the respective chromosomes (each ERCC transcript is annotated as an individual chromosome), there is no signal to be mapped beyond these ends. For Fig 1H, segregated TSS and TTS scattered throughout the chromosomes are used to map the coverage on a base-pair coordinate without any meta scaling. To make the data presentation more clear, we now show that when zoomed in on the TSS and TTS (new S1K Fig), the profile in Fig 1H shows a similar trend as seen for Fig 1D. Thanks for pointing out the mistake regarding the heatmaps in the figure legend. We have corrected the figure legends now (lines 1042-1044).

R1.c From Figure 2f the authors claim there is a convergence between the short and long read assemblies because there is an agreement of ~20%. To me an agreement of 20% is very low and would signify the opposite of a convergence. It could be there is some missing context here that explains why 20% is a convergence, if so the authors should include this.
While we agree that 20% convergence would be very low, we note that the set criteria (exact match of entire transcript) is very stringent. When a more relaxed criterion (multi-exon with at least one junction match) is also taken into account, the agreement is 50-55%. However, as the reviewer suggested, we included a new analysis to provide the context. We report that 70-90% of transcripts show overlap between short- and long-read assemblies (new Fig 2F). Then we provide the fine-scale comparison of transcript structures (Fig 2G). Taking the reviewer’s point into account, we removed the statement for “high convergence” (lines 285-295).

R1.d In Figure 2g a line is drawn signifying when the short-read coverage is close to the long-read coverage. The authors should include how this equal coverage is defined as there are several possible interpretations such as the same number of reads, the number of mapped bases, the average coverage across all bases etc.
The equal coverage is defined as the number of mapped bases. We have now made it clear in the corresponding figure legends (lines 1076-1077).

R1.e Also in Figure 2g it is claimed that depth is important however when comparing 100% sampling versus 10% where the coverage is similar, there is not a lot of difference in the measured metrics excluding the containment of the long-read, and therefore coverage does not seem to be a reason why short and long read assemblies are different. The authors should clarify this claim or better explain why they believe depth is important based on these results.
We appreciate the reviewer’s attention to detail. We agree that many of the class codes don’t show drastic change after the point of equal coverage. We addressed this point in two ways: first, we included another data point with 5% coverage that emphasizes change in measured metrics up to the 10% coverage point (new Fig 2H); second, we revised the text to reflect the saturation post 10% coverage and possible reasons for it (lines 296-301). We observed that the total number of transcripts assembled

in the short-read assembly increases up to 10% coverage with minimal change thereafter (new S2I Fig). So, the point of equal coverage denotes the maximum limit of coherent assembly for long-read assemblies as well as saturation of the total number of transcripts assembled in the short-read assembly. Finally, we softened the emphasis on coverage being the only factor dictating the divergence between the two platforms (lines 310-312).

R1.f Figure 5c should be made clearer in the legend that each point represents only the ERCCs at that concentration level as it can be hard to interpret the figure without realizing that.
We have revised the legend to make it clear (lines 1137-1138).

---**Reviewer #2**---

Reviewer #2: This manuscript by Kainth et al proposes a methodology to combine short (Illumina) and long (Nanopore) reads transcriptome sequencing to improve the identification of transcripts. The authors show that Nanopore sequencing has a high rate of false antisense transcripts due to the lack of strand information of the Nanopore reads. They develop an analysis strategy that is able to correct the strand mapping of these reads. Then they combine short-reads and stranded long-reads based transcripts inferred by StringTie to provide a consensus transcriptome prediction (TASSEL) . The rationale of this approach is that the higher sequencing depth of the short reads, in comparison to long reads, make it possible to identify more transcripts. They compare their approach to StringTie mix, FLAIR and Bambu and use as benchmarking dataset the Lexogen ERCC set. Finally, they apply their method to the identification of chromatin enriched RNAs (cheRNAS) in two cell lines HAP1 and mouse HL1.

This work has two components, the development of TASSEL as a tool for the reconstruction of transcripts using long and short reads, and the analysis of cheRNAs using this method.

R2.0 The false identification of antisense transcripts due to the lack of strand information of Nanopore reads is a known limitation of the technology and the development of a strategy to mitigate this is relevant contribution. However, their approach is based on the identification of cDNA primers, which according to their result was only successful for about half of the reads. This should be highlighted in the discussion.
We thank the reviewer for acknowledging the relevance of our stranding pipeline. Although the extent of stranding is not 100%, it is still substantially higher (60-75%) than the currently available method (S3A Fig). We also note that the removal of the unstranded reads does not seem to hamper the sensitivity of transcript detection or assembly. On the contrary, new analyses on spliced sequin spike-in standards show that SLURP stranding leads to an overall increase in the sensitivity and precision of transcript assembly (new Figs 3F and 3G). We reason that this happens due to the removal of the underlying noise caused by misaligned reads that not only reduces the confidence of mapping in the correct orientation but also creates discontinuity of stranded reads for intact transcript assembly. This is supported by new examples in Figs 3H, S3E and S3H where removal/stranding of misaligned reads led to assembly of intact transcripts. We have also added treatment of the lack of complete stranding by our methodology in the discussion section (lines 587-592).

Moreover, I am concern with several of the aspects in the strategy they took to benchmark their approach.

R2.1 The benchmarking is done based on ERCC, which are synthetic, not spliced transcripts with no alternative isoforms and they conclude on performance based on this data. However, not spliced, single isoform genes are rare in the human genome, therefore the good performance of their approach on the ERCC data cannot be extrapolated to the broad of the human transcriptome where alternative splicing and the presence of similar isoforms in each gene, is abundant. The SIRVs spike-in sets of Lexogen, which contained multiple alternative isoforms of the same genes, are a better benchmarking dataset which should be used. Otherwise, the authors should clearly state that they approach has only be validated on mono-exon genes.

We wholeheartedly agree with the reviewer that the ERCC spike-in standards may not reflect the complexity of spliced transcripts typically found in real transcriptomes. In the revised manuscript, we have highlighted this limitation in lines 217-220. Inspired by this suggestion, we sought to test SLURP and TASSEL on alternatively spliced spike-in standards such as SIRV or sequins. We reasoned that the sequins are more suitable than SIRVs to address reviewer's suggestion of benchmarking with multi-exonic complex spike-in standards as sequins have higher range of length distribution (280-7000bp for sequin versus 160-2493 bp for SIRV), a greater number of transcripts (165 transcripts and 82 genes in sequin versus 69 transcripts and 7 genes for SIRV) and higher dynamic range of abundance (7.62×10^6 for sequin vs none for SIRV). Importantly, there are publicly available long-read and corresponding short-read datasets for the sequin spike-in standards from the SG-Nex consortium for us to test SLURP and TASSEL to address this critical point. We report that SLURP not only corrected the strand of transcript assembly but also improved the overall sensitivity and precision of the sequin transcriptome (new Figs 3F and 3G). We also evaluated TASSEL on the sequin transcriptome – when provided with 100% of sequin annotation, TASSEL showed higher sensitivity than other methods (new Fig 5E). We further tested the *de novo* transcript assembly capabilities of TASSEL by providing only 50% of the sequin annotation (new Fig 5F). Here again, TASSEL assembled the highest number of correct transcripts from the omitted portion of the annotation.

These multiple lines of evidence on two sets of distinct spike-in standards indicate that TASSEL provides improvement in transcript discovery as well as assembly of complex transcripts.

R2.2 The authors use multiple data sources to validate TTS and TSS of their predicted transcript models and they show that TASSEL recover those better than other approaches and conclude that the transcripts are better assembled. Again, this conclusion is valid for monoexon genes but not for alternatively spliced transcripts, where multiple combinations of intron chains and TSS/TTS are possible.

In addition to the new analyses about spliced assembly in general detailed in the above R2.1 response, we have now performed new analyses with a subset of spliced sequin transcripts with alternative TSS or TTS to demonstrate increased sensitivity and precision of transcript detection for the class of transcripts that the reviewer suggested (new Fig S3D and S3E).

We would also like to emphasize that our findings from the comparison of TASSEL with StringTie Mix to detect transcription start and termination sites in the human transcriptome in our HAP1 dataset is actually representative of the greatest isoform variation (ref TTS/TTS variation).

Indeed, previous studies report on alternative TSS and TTS being a larger source of isoform variation than splicing across human tissues (Reyes et al., *Nucleic Acids Research*, 2018). The choice of TSS and TTS can not only dictate the fate of transcript function (Carlos Alfonso-Gonzalez, *Cell*, 2023) but also enables effective molecular manipulation for functional studies (Gilbert et al., *Cell*, 2014; Radzisheuskaya et al., *Nucleic Acids Research*, 2016). The high prevalence of TSS and TTS variation argues that our ERCC analyses (Fig 5D) remain a valid benchmarking method. Therefore, we retained our original analyses alongside the new data. Here we used multiple molecular features (H3K4me3, Pol II occupancy and PRO-seq) measured in the same cell line to probe the termini of transcripts from each assembly. All those analyses (Figs 5H, 5I and 5J), effectively done on complex mono- as well as multi-exonic transcripts, show that TASSEL performs better than StringTie Mix. We provide additional evidence that consensus human transcripts (mono- as well as multi-exonic) in TASSEL provide better enrichment of TSS and TTS than those derived from the short- or long-read alone (new S5G and S5H Figs). These features of TASSEL are now discussed in the discussion section (lines 424-429 and 642-645).

R2.3 Related to the two previous comments. It is unclear how their method performs on multi-exons genes with several alternative isoforms, and this should be specifically addressed.
We agree with the reviewer that spliced isoforms present a challenge for correct transcript assembly not captured by mono-exonic ERCC standards. To address this point, new analyses with sequin spike-in standards assuage this concern as 157 out 165 sequin transcripts are multi-exonic and 156 out of 165 sequin transcripts have a spliced isoform. Using these standards, we show that SLURP as well TASSEL improve transcript assembly as compared to other methods (new Fig 3F, 3G). To illustrate this point, we show an example of *R1_22* sequin spike-in standard transcript which is a multi-exonic transcript with two spliced isoforms (new Fig 3H). The original unstranded long-read assembly fails to assemble any of the two isoforms whereas use of SLURP led to assembly of both isoforms in the correct direction with exact exon structure as the reference transcript. In a larger scope analysis, TASSEL also leads to better assembly of the sequin transcriptome (new Fig 5E, 5F and 5G). Furthermore, we now show that TASSEL refines both mono- as well as multi-exonic cheRNA in our HAP1 dataset (new Fig S6E).

R2.4 The authors claim that a problem of long reads data for transcript assembly is the lower sequencing depth, and they show several analyses to proof this. Their long reads dataset has 1-3 million reads. However, much higher sequencing depths (up to 10-20 million reads) on one MinIon are now possible with Nanopore sequencing. Will TASSEL still provide an advantage in this scenario?

This is an interesting question. In addition to artificial drop out methods used in Fig. 2H, the reviewer proposes using deeper long-read datasets for orthogonal evaluation of the assembly performance as a function of the relative depth of the two datasets. After an extensive search, we were unable to locate any direct-cDNA datasets of that depth accompanied by corresponding short-read data to enable the analyses exactly as proposed. However, we were able to find a long-read dataset (~7 million reads) from the SG-Nex consortium to benchmark SLURP and TASSEL on sequin spike-in standards that is 3-4 times deeper than our datasets. Our new analyses of three paired datasets used to benchmark TASSEL covers a wide range of relative depth (new S5K Fig). As noted in point R2.1 and R2.3, we find that both SLURP and TASSEL are equally effective on this deeper dataset (Figs 3G, 3H, S3D,

S3E, S3F, 5E, 5F, 5G, S5B and S5F). The prime reason for this is that the use of SLURP not only orients the primary sequencing reads in the correct direction, it also removes the potentially ambiguous reads. This dual corrective action culminates to an increase in the proportion of correctly matched transcripts as well as reduction in the "noisy" erroneous reads, irrespective of the depth of the sequencing run.

R2.5 Figure 5. Unclear how the sensitivity with the entire transcriptome as the ground truth is not known. The methods section indicates that this analysis is taking as ground truth the genome annotation, which is not an accurate option, as genome annotation is incomplete even in extensively studied genome as human, and not all annotated genes or transcripts are present in each cell line or tissue Therefore, sensitivity and precision cannot be calculated in this way. Either these metrics are re-named to reflect that the ground truth is unknown, of a methodology is used that contains the ground truth, for example, simulated data, spliced SIRV or a subset of manually curated transcripts.
This is an excellent point. While we agree that the human reference annotations do not provide the perfect "ground truth" for sensitivity and specificity measurements, we do feel that it is important to test assembly methods on mammalian transcripts as they are not only more complex than any spike-in controls but also provide "real-world" information about the efficacy of these tools. Thus we have retained these analyses in the manuscript, but have provided more explicit explanation of their clear limitations. Specifically, in places where we used genome annotations as the reference point, we have added a cautionary statement to reflect inherent ambiguity of mammalian reference genome annotations in specific cell types as highlighted in this critique (lines 246-249, 367-370). To make these limitations clear in the corresponding figures, we added the word "apparent" to sensitivity and precision measures to indicate imperfection of the reference annotations for the given cell types (S2A Fig and S5C Fig).

Most importantly for this point (and related to the above points), we have now benchmarked specificity and sensitivity using alternatively spliced spike-in standards and rely more heavily on these analyses to evaluate the performance of assembly pipelines. This new benchmarking of SLURP and TASSEL based on the reviewer's suggestion of spliced spike-in controls is a much better metric than transcriptome matching as the ground truth of their transcript structures is known. With these new sequin spike-in data, we performed our analyses in two modes: one in which we provided 100% of sequin annotations to transcript assembly programs to test how well these programs can assemble the known transcripts, and a second in which we provided only 50% of sequin annotations to test *de novo* assembly capabilities of assemblers. In both scenarios, we report that TASSEL outperforms other methods in terms of assembling highest number of correct transcripts (Figs 5F and 5G). We hope that these changes provide the reader context for the analyses presented, and place more emphasis on the sequin and ERCC standard analyses of specificity and sensitivity.

Regarding the analysis of the cheRNA fraction of the two cell lines, I have some concerns.

R2.6 Only the Chromatin fraction was sequenced by long reads. Since the manuscript indicates a low agreement (20-25%) between short and long reads transcripts, there is a risk that the cheRNAs detection is highly biased by the transcript detection properties of the long reads technology. In fact, their analysis indicates that only 20-40% of the chromatin genes obtained with short reads are covered by the long reads. This is attributed to the lower sequencing depth of the long reads but posible other

capture issues might occur. Actually, Figure 2C shows that the great majority of the long reads transcripts are intron retention, which is not the case for the short-reads data. More clarity if needed to understand how the sequencing technology used in each fraction is impacting the biological conclusions of the analysis.
We apologize for the lack of clarity on this point in our original submission. We have now clarified the relevant text (lines 140-145 and 955-967) and added new analyses (new Figs 2F, 6C, S5D) to address this concern. We defined "cheRNA" on the basis of the ratiometric enrichment of a given transcript in the chromatin fraction as compared to the nucleoplasmic fraction only from short-read sequencing as it has higher depth and is consistent with our previous analyses (Werner & Ruthenburg, *Cell Reports* 2015; Werner et al., *Nat Struct Mol Bio* 2017; Yang et al., *eLife* 2017; Sun et al., *PLoS Computational Biology* 2020). The long-read sequencing was performed only for the chromatin fraction for comparison with short-read datasets from both fractions and subsequent refinement. First, we compared *all transcripts* in the chromatin fraction for short- and long-read datasets to ascertain the strengths and weakness of the two platforms (Figs 1 and 2). As the reviewer pointed out, we discovered platform-specific limitations; for example, a substantial fraction of long-read transcripts represent intron retention. A possible reason for this could be artifactual segmentation of long-read transcripts due to low coverage and/or internal priming events (as the reviewer alluded to in point R2.f). We have now added this discrepancy in the revised manuscript (lines 262-264, 293-295, 568-569).

Then we attempted to remediate the platform-specific limitations by integrating short- and long-read assemblies using TASSEL. Here again, merging is performed on *all transcripts* in the chromatin fraction from short- and long-read datasets. To address the reviewer's concern, we tested the prevalence of intron-retention transcripts in the TASSEL transcriptome. As shown in the new S5D Fig, we report that TASSEL leads to a substantial reduction in the proportion of intron-retention transcripts. We reason that this happens due to the correction and consolidation by the short-read dataset.

Finally, we tested TASSEL on "cheRNA" (Fig 6). For this we only compared "cheRNA" transcripts from the chromatin fraction of the short-read dataset, which are only a subset of total chromatin fraction transcripts (as defined by their relative enrichment in the chromatin vs nucleoplasm fraction; Fig 6A). We have added a new figure to test the broad overlap between "cheRNA" genes and those obtained from original long-read genes (new Fig 6C). More than 75% of "cheRNA" genes from the short-read dataset had an overlapping gene (any overlap) in the long-read datasets. We agree with the reviewer that the convergence is low for higher extent (90-100%) of overlap. Taking the reviewer's suggestion into account, we have now discussed this lack in convergence possibly due to the platform-specific variations we found earlier (lines 512-514). Finally, similar to *all transcripts* (Fig 5), we tested if TASSEL can alleviate this disparity to refine the "cheRNA" transcripts as well. As shown in Fig 6D, 6E, 6F and 6G, use of TASSEL refines the cheRNA transcripts by these metrics.

R2.7 When comparing chromatin and nuclear fractions, the transcriptomes have to be merged. It is not clear how incomplete transcripts or unprocessed transcripts are considered.
To address this point, we have now added a detailed description of the procedure that we used in the methods section (lines 952-967).

In brief, the transcripts from chromatin and nuclear fractions (as assembled by StringTie on the respective alignment files) are merged with default settings of StringTie --merge. This is a standard

way to create a high-confidence unified set of non-redundant transcripts across the samples used for any type of analyses that require differential gene expression of known as well as *de novo* transcripts (Pertea et al, *Nature Protocols*, 2016). (The way we have handled ratiometric comparisons of the transcripts in the two fractions is using differential expression pipelines [Werner & Ruthenburg, *Cell Reports* 2015; Werner et al., *Nat Struct Mol Bio* 2017; Yang et al., *eLife* 2017; Sun et al., *PLoS Computational Biology* 2020]). This merging rectifies any incomplete transcript in a given sample if it detects a corresponding complete transcript in another sample. This unified and consolidated pool of transcripts across the chromatin and nuclear fractions was then used as a reference transcriptome to re-estimate transcript abundance in each of the replicates of both fractions. The re-estimated abundance was then used with DESeq2 to calculate statistically significant ratiometric enrichment of a given transcript in the chromatin fraction to deem as cheRNA. Therefore, the use of unified set of transcripts created by StringTie --merge to calculate ratiometric enrichment avoids the influence of sample-to-sample background heterogeneity as all the samples are re-evaluated against a common template. These details are now added in the methods section.

Other points of revision are:

R2.a Authors conclude from Figure 2B Good correlation between replicates in long reads sequencing. However, the plot shows that correlation at the m[e]dium/low expression range might not be. Using one correction value along the whole expression range is misleading as the highly expressed genes pull the correlation coefficient to high values. This analysis should be revisited.

We agree with the reviewer that the plot shows lower correlation for low expression genes. This is consistent with higher counting error of lower signals observed in many cases of genome-wide datasets due to increase in "noise" as one approaches the limit of detection. The overall correlation coefficient falls well within the range correlation coefficients observed for long-read sequencing in the literature (e.g., Response Figure 1; from Chen et al., *Nature Methods*, 2023).

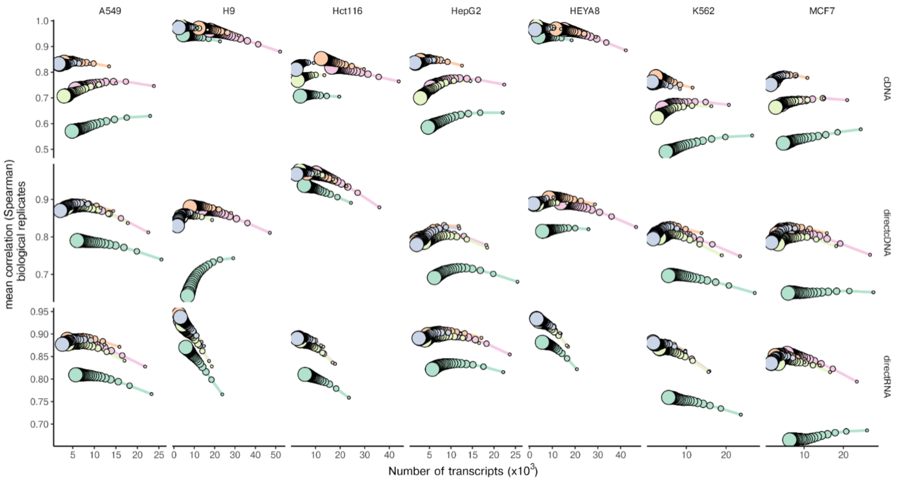

Response Figure 1: Correlation coefficient of long-read replicates for different cell lines and library preparation methods at different levels of precision (indicated by size of the marker). Taken from Chen et al., *Nature Methods*, 2023, supplementary figure 6.

As per the reviewer's suggestion, we have added an appropriate statement to indicate divergence across replicates for low-abundance transcripts (lines 251-253).

R2.b I disagree that Figure 2E shows a high correlation between long and short reads data. Moreover, it is not clear how the pairing on long and short transcripts is done for this graph. Are there only exact match considered? How is redundancy handled, i.e. when one long reads transcript is a perfect match by multiple short reads transcripts or vice versa.

We regret that our original text was unclear on this point. We agree with the reviewer's interpretation of the lack of overall correlation between short and long-read transcript abundance estimation, particularly at the lower depth range. We note that this is consistent with higher sensitivity of short-read sequencing for low abundance transcripts (Fig 2A, S2B). Accordingly, we rewrote the corresponding section to better reflect the lack of correlation for medium to low abundance transcripts (lines 279-284).

We have now added more detail in the methods section describing the comparison of short- and long-read transcript abundance (lines 860-868) and bolstered the corresponding figure legend (Fig 2E). In brief, for comparing abundance of transcripts and genes in short- and long-read samples, StringTie-assembled gtf files of long-read samples (2 replicates) and short-read samples (3 replicates) were merged using stringtie --merge to obtain a unified non-redundant pool of transcripts across short- and long-read samples. This merge function also effectively consolidates overlapping (e.g., when one long-read transcript matches multiple short-read transcripts), truncated or incomplete transcripts in a sample if it finds evidence of a complete transcript in another sample. This merged transcriptome gtf file was then used as a reference for re-estimation of transcripts in each of the samples using stringtie estimation mode with -e option. Then, average transcript and gene abundance (Tags per million, TPM) of every transcript and gene was calculated across the replicates for short-read and long-read samples. These average TPMs were compared to test the correlation between short-read sequencing and long-read sequencing (Fig 2E).

R2.c The authors conclude on the absence of length quantification biases for short and long reads sequencing from ERCCs, which is a reduced set of transcripts. Can the same conclusion be extracted for the whole transcriptome?

It is a fair point that ERCC standards do not reflect the complexity and length spectrum of mammalian transcriptomes. In the revised manuscript, we added a new analysis to test coverage of known human genes in our short- and long-read datasets (new S1G Fig) where we observed that the coverage per kb per million mapped reads is independent of the length of genes. We have also included the corresponding analyses with sequin spike-in standards as they encompass more complexity (in terms of splice variants and number) and a larger length distribution range than ERCC standards (0.2-2kb for ERCC vs 0.3-7kb for sequins). Our analyses with sequins (new S2B Fig) show absence of length quantification biases for short- and long-read sequencing on a size regime that encompasses the median human transcript length (Lopez et al., *Frontiers in Genetics*, 2021). However, we have included a cautionary statement (lines 246-249) for mammalian transcriptomes for which the ground truth for each cell type is not known.

R2.d I do not think that agreeing in a high convergence between short and long reads assembly can be drawn when only 20-25% of the long-reads transcripts are matched by a short-reads transcript (line 261).

While we agree that 20% convergence would be very low, we note that the set criteria (exact match of entire transcript) is excessively stringent to effectively convey the overlap to the reader. We have included a new and more direct analysis that indicates that 70-90% of transcripts show overlap between short- and long-read assemblies (new Fig 5F) and provide a fine-scale comparison of transcript structures (including this exact match subset). Taking the reviewer's point into account, we removed the statement for "high convergence" (lines 285-295).

R2.e The authors found a very interesting artifact in the cDNA library preparation which they address in their analysis. However, this is not the only artifact of cDNA libraries. Other are RT switching (which is similar but only skips a part of the transcript) and intrapriming, where the oligodT primes at A stretches different from the polyA, for example at introns. I wonder how their pipeline handles these types of cDNA library preparation errors.

As the reviewer points out, internal priming artifacts are endemic to any 3' end-targeted oligo-T-based library preparation method. However, they remain a challenge to distinguish from other confounding factors: *i.*) high prevalence of *bona fide* alternative poly-adenylation, *ii.*) RNA fragmentation during library preparation prior to 3'-end pA tailing, and *iii.*) pore slipping prior to data collection during nanopore sequencing. As a rough way of estimating the collective contribution of these factors to internal mapping of reads (absent any alternative polyadenylation), we calculated the percent of reads that do not originate from the known 3' ends of the two most abundant ERCC transcripts. As the precise origins of these internally mapped reads are uncertain and there is no way of testing this in a transcriptomic dataset without the ground truth, we are hesitant to include this analysis in the manuscript.

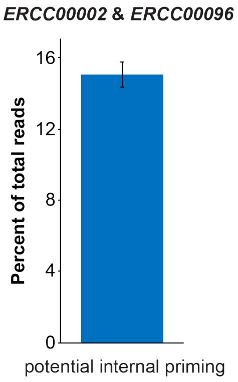

Response Figure 2: Percent of total reads not mapping to the 3' end of the ERCC reads.

However, as the reviewer pointed out (R2.6), we observed that a substantial fraction of transcripts in our original long-read dataset were deemed "intron retention". Similar levels of such transcripts were also detected in other studies (Shumate et al., *PLoS Computational Biology*, 2022). Perhaps, the origin of such transcripts can partially be due to the internal priming of oligo-dT primers in the A-rich regions of introns (La Manno et al., *Nature*, 2018). Our short-read assemblies had far fewer of these transcripts as our short-read libraries were prepared using random hexamers instead of oligo-dT primers. Therefore, we speculate that our TASSEL merge also leads to correction and consolidation of such long-read transcripts by short-read transcripts (new S5D Fig). In support of this interpretation, there are no ERCC standards that are detected for which TASSEL does not accurately capture the 3' end (S5A Fig). We have added these points in the discussion section (lines 654-658).

R2.f When comparing their method to Bambu and FLAIR, I believe that only Long reads data is used for these comparing approaches. I am not sure about Bambu, but I believe FLAIR can integrate short reads data as well. I would be fairer to compare their method to FLAIR using both short and long reads data.

This is a great point. Per this reviewer's suggestion, we integrated the corresponding short-read dataset with FLAIR. As shown in the new Fig 5E, integration of short-read dataset with FLAIR still underperforms StringTie Mix as well as TASSEL, but this is a more objective comparison now.

R2.g I found figures 1F and 1D for the long reads data, give contradictory results. Figure 1F suggests a general lack of full-lengthness for long reads sequencing, as more coverage is seen at the transcript ends. However, the data from the ERCCs do not show that. Is there any explanation for this result?

We value the reviewer's keen eye. The discrepancy arises due to a substantial difference in the length distribution of ERCC genes versus known human genes. The ERCC transcripts range from 0.2-2kb with a median of around 0.9 kb which is similar to the median length of long reads in our dataset (1 kb, Fig 1B). However, many human genes are much larger than the median long read length. Due to this reason, when plotted on a scaled metagene, the coverage plot shows a decay in the gene body. This is supported by our new analysis restricted to human genes that are between 0.2-2kb in length (new S1I Fig). The resulting profile of long-read coverage on the length restricted human genes looks similar to that of the ERCC genes.

---**Reviewer #3**--

Reviewer #3: The manuscript "Merging short and stranded long reads improves transcript assembly" describes the development of tools to overcome the problem of strand assignment of long reads and to combine long and short reads to improve the resolution of transcript models, and then applies the tools to the annotation of cheRNA transcripts. The manuscript is very clearly written and the analysis and presentation of results similarly thorough and clear. There is a very comprehensive introduction to the use of long transcriptomic data in transcript annotation and useful investigation of the artefacts that can be identified. The final study of cheRNAs demonstrates the utility of the tools described earlier.

R3.1 There is a lack of discrimination between spliced and unspliced/monoexonic lncRNAs. While the manuscript indicates that it is the annotation of spliced transcripts that is being addressed eg Figure 2C it seems that much of the benefit of the tools described relates primarily to monoexonic lncRNAs. For example, while SLURP demonstrably improves the strand assignment for long reads, this is less of a problem for spliced transcripts which can be stranded using splice site information from aligned reads. Similarly the cheRNA study, where SLURP and TASSEL improve on the transcript annotation over methods using unstranded long reads or short reads alone, appear to be focused on the annotation of monoexonic lncRNAs.

We thank the reviewer for pointing out some of the limitations of our original analyses especially with mono-exonic ERCC spiked-in standards. In the revised manuscript, we provide several lines of evidence to show that both SLURP and TASSEL also improve multi-exonic transcript assembly. To this end, we used datasets with multi-exonic sequin spike-in standards with spliced isoforms to benchmark the efficacy of SLURP and TASSEL. Using these multi-exonic standards, we show that SLURP: *i.*) reduces the proportion of transcripts mapping to the wrong strand of the reference annotation (new Fig 3F), *ii.*) increases the overall sensitivity and precision of transcriptome assembly, independent of the assembly program (new Fig 3G), and *iii.*) rectifies erroneous assembly of a multi-exonic transcript with spliced isoforms (new Fig 3H, S3D). Then we probed the efficacy of TASSEL with these multi-exonic

standards. We find that TASSEL: *i.*) assembles the highest number of correct known transcripts (new Fig 5F), and *ii.*) assembles the highest number of correct *de novo* transcripts (new Fig 5F).

We would also like to draw attention to our original and new analyses of SLURP and TASSEL on our HAP1 dataset. We showed that SLURP rectifies erroneous mapping of long reads to the human transcriptome, which include both mono- and multi-exonic transcripts (new Fig 3I). Taking the reviewer's suggestion into account, we have now added an example where SLURP correctly assembles a known multi-exonic human transcript where the original unstranded reads failed to do so (new S3H Fig). We agree with the reviewer that splice site information from aligned reads can theoretically be used for strand orientation, but the high error rate in long-read sequencing would be a major bottleneck in doing so. This is highlighted in the example we have provided now where the assembly from unstranded long reads led to creation of fictitious exons as well as omission of *bona fide* exons (new Figs 3H and S3H). Additionally, use of splice sites to orient reads would be limited to transcript models with known and canonical splice-sites which will hinder the assembly of novel transcripts. Recent studies also suggest that many splicing events occur independent of canonical intron/exon and branch-point definitions (Herzel et al., *Nature Reviews Molecular Cell Biology*, 2017; Zeng et al., *Molecular Cell*, 2022). As we show in the revised manuscript, SLURP is not dependent on any *a priori* information of splice sites or accompanying short-read datasets. Similarly, we tested TASSEL to demarcate transcript ends on the basis of multiple molecular features (H3K4me3, Pol II occupancy and PRO-seq). All those analyses (Figs 5H, 5I and 5J) derived from the same cell line, effectively done on complex mono- as well as multi-exonic transcripts, show that TASSEL performs better than other methods. Furthermore, per the reviewer's suggestion, we have now included an example where TASSEL correctly assembles a known spliced human transcript where the original short- or long-read dataset failed to do so (new S5I Fig).

Based on these multiple lines of evidence, we propose that SLURP and TASSEL can be used for improved assembly of complex multi-exonic transcripts.

R3.2 It would be very useful if this issue could be discussed in the manuscript with perhaps some analysis provided in the supplementary data to assess the impact of SLURP and TASSEL on monoexonic vs multiexonic lncRNAs.
We thank the reviewer for this thoughtful suggestion. In addition to the new detailed analyses of multi-exonic sequin spike-in standards and human transcripts (discussed in point R3.1), we have now included a new analysis to show that assembly of both mono- as well as multi-exonic cheRNA is improved by TASSEL (new Fig S6E). We have also highlighted these points in the results as well as the discussion sections (lines 110-113, 355-365, 424-430, 523-524, 642-645).

R3.3 There is also some scope to add more explanation and analysis regarding the annotation of TSS and TTS. Was any testing done of the relative contribution of long vs short reads to the ability of TASSEL to corectly identify transcript termini? For example, analysis earlier in manuscript presents the coverage of transcripts by both datatypes, but no specific analysis is presented eg what happens to the H3K4me3 occupancy, Pol II occupancy and PRO-seq signal plots when the reads from each data type are removed? Does all the TSS signal come from long reads?
In response to this point, we have now performed detailed analyses of the contribution of each of the individual components of TASSEL. Using the complex multi-exonic sequin spike-in dataset, we show

that TASSEL provides higher sensitivity of transcription detection than short- or long-read alone (new Figs 5F and 5G). In the new Fig. S5F, we report on the equivalent relative contribution of short- and long-read transcriptomes to the TASSEL transcriptome. Furthermore, we find that the consensus transcripts in the TASSEL transcriptome evince better enrichment of TSS and TTS (S5G Fig) as well as H3K4me3 occupancy, Pol II occupancy and PRO-seq signal (S5H Fig) than the transcripts obtained from either of the short- or long-read transcriptomes alone.
