# Supplementary material for: Merging short and stranded long reads improves transcript assembly
Source: PLoS Comput Biol. 2023 Oct 26;19(10):e1011576. doi: 10.1371/journal.pcbi.1011576 (PMC10629667; doi:10.1371/journal.pcbi.1011576)
Supplement: Attachment — Submitted filename: KainthHaddad_ResponseLetter_R2.pdf [file pcbi.1011576.s011.pdf]

I appreciate your thorough response to the prior reviewers' comments and, in particular, the addition of benchmarks with the sequin spike-in RNA standards.
We thank the editor for acknowledging our efforts to address what was the salient concern raised by all the reviewers.

However, due to challenges associated with reviewer availability, an additional reviewer (Reviewer 4) was recruited to review the revised manuscript, and Reviewer 4 has raised some significant new concerns. In particular, Reviewer 4 has concerns about the novelty, need for, and evaluation of the computational pipeline in light of the existence of the ONT pychopper tool and other more recent methods that can clean and orient ONT reads. It is unfortunate that these major concerns are being raised at this stage of the review process, but they must be addressed before a decision is made regarding the suitability of this work for publication in PLoS Computational Biology.
It is indeed unfortunate that the original reviewers 2 and 3 were not available to assess the manuscript revisions that were performed to address their concerns. Nonetheless, we genuinely believe that many of their suggestions improved our manuscript.

In the second revision of the manuscript, we have now added new analyses to address reviewer 4’s concerns regarding potential redundancy with Pychopper and all other methods suggested by the reviewer that “can clean and orient ONT reads”. The only exception is TALC, which we could not get to run and found its authors unresponsive about these code issues. Remarkably, SLURP outperforms Pychopper and ReorientExpress in terms of sensitivity and precision of transcript assembly as well as computational overhead (new Figs 3E, F, S3E, F, G, H, I, J and Response Figure 1). We have also clarified how the palindromic artifact investigated in this study is not reported for the ONT platform in the literature that we could find (certainly not in the references cited by reviewer 4) – this is completely distinct from closed circular sequences (CCS) reads of the PacBio platform that are a normal part of library preparation for that platform. Finally, taking all of reviewer 4’s suggestions into account, we report that TASSEL outperforms all other stand-alone (Bambu, FLAIR, isONform, IsoQuant, Mandalorion, RATTLE, RNA-bloom2, StringTie2, TALON and TAMA) and hybrid (FLAIR with short-read correction, StringTie Mix, RNA-Bloom2 with short-read correction) transcript assembly pipelines that we tested (new Figs 5B, 5E, S5C, S5E and Response Figure 3), making a strong case that our pipelines represent an important advance for the field.

As the main technical points of our paper are SLURP (our stranding preprocessing method for ONT cDNA-based long read RNA libraries) and TASSEL (our hybrid assembly method that combines stranded ONT long-read and short read Illumina data), we feel that benchmarking other non-hybrid assembly tools versus our pipeline is largely beyond the scope of this work. Nevertheless, in addition to the six pipelines we had previously, we tested *all* the pipelines suggested by the reviewer to benchmark

SLURP and TASSEL. Taken together, we hope that you agree that SLURP and TASSEL are valuable tools with the potential to markedly improve transcript assembly using long-read ONT and hybrid methods.

Reviewer #1: In this response the authors have addressed all major and minor issues I raised after reading the initial submission. The addition of the analysis using sequins was a great addition to the manuscript and strengthens many of the claims the authors make with TASSEL. I also appreciate the significant developments TASSEL made, enabling its use on PCR cDNA ONT samples.
We are grateful for reviewer 1's input in the first round of reviews. We note that while reviewers 2 and 3 are not available to assess the revised manuscript, they shared many of the concerns with reviewer 1, especially for enhanced testing of our pipelines on a more challenging set of gold standard transcripts. We are gratified that this reviewer believes that we addressed the major concerns raised in the first round of reviews.

Reviewer #4: The authors propose a pipeline TASSEL to assemble transcripts from short and long dRNA and cDNA sequencing reads. The pipeline contains standard steps such as orienting the reads (with their tool SLURP), aligning the oriented reads with minimap2, and assembling transcripts with StringTie2.
We are grateful for this reviewer's expertise and benchmarking suggestions which we feel have improved the paper, particularly the Pychopper comparison. The specific concerns are addressed below.

The authors forget to perform standard primer trimming with pychopper (standard to ONT long-read processing), which is probably also why FLAIR performs badly, as it expects preprocessing.
We thank the reviewer for bringing Pychopper to our attention and providing it as a key comparison point to benchmark SLURP. Upon initial inspection, the underlying principle of using primer sequences to orient reads seems similar for Pychopper and SLURP. To our surprise, SLURP performed significantly better than Pychopper (detailed in section R4.1 below), perhaps due to the way the sequences are searched within the reads and/or tolerance of mismatches.

While Pychopper is used in many ONT long-read RNA-seq papers, there are many seminal studies that do not, arguing that it is not used as an obligate processing step. For example, the following studies did not use Pychopper to process reads: Kovaka et al., *Genome Biology*, 2019 (for StringTie2); Broseus et al., *Bioinformatics*, 2020 (for TALC); Ewels et al., *Nature Biotechnology*, 2020, (for Nanoseq pipeline); Lebrigand et al., *Nature Communications*, 2020; Sousa-Luis et al., *Molecular Cell*, 2021 (for POINT-Nano); Shumate et al., *PLoS Comp Bio*, 2022 (for StringTie Mix); Prjibelski et al., *Nature Biotechnology*, 2023 (for IsoQuant); Chen et al., *Nature Methods* 2023 (for Bambu). Even widely used software can often be improved upon, and indeed we feel

our direct comparisons between SLURP and Pychopper in the revised manuscript demonstrate SLURP embodies such an improvement (see below section R4.1).

It is indeed true that FLAIR performs poorly without preprocessing. In the revised manuscript, we provided FLAIR with either SLURP- or Pychopper-processed reads and report that SLURP improved FLAIR's performance substantially more than Pychopper (new Fig S3H). To incorporate this helpful suggestion, for all subsequent uses of FLAIR in the paper (new Figs 5B, 5C, 5E, S5B and S5E), we have now used SLURP to preprocess (because in these instances as well, FLAIR performed better with SLURP than with Pychopper).

The main findings presented, such as "long-read transcript assembly lacks depth and strand-of-origin" and "We also discover a cDNA synthesis artifact in long-read datasets" are therefore not true (pychopper can be used to fix both of these claims). Consequently, SLURP may be redundant compared to the previously designed tools for the same purpose.

For *"long-read transcript assembly lacks depth and strand-of-origin"*: The typical cDNA-based long-read RNA-seq libraries inherently lack strand of origin information as opposed to the stranded short-read libraries (prepared by methods such as NEB's dUTP-based library prep). To address the reviewer's concern, we have improved the accuracy of the wording to reflect the same (lines 23-24, 86-87, 320-321). That stated, we note that "long-read transcript assembly lacks depth and strand-of-origin" is not a major finding of our paper, but rather a known challenge, that part of our paper addresses.

For "*We also discover a cDNA synthesis artifact in long-read datasets*": While there are many known artifacts, the one reported here is to our knowledge novel, and the literature cited by the reviewer does not provide support that this particular palindromic artifact was previously known (see point R4.3 below). While Pychopper is sensitive to the presence of forward and reverse complements of each of the primers, Pychopper does not fix these reads in its "full length reads" output (aside from discarding them entirely). Furthermore, the presence of forward and reverse primer sequences within a given read do not necessarily indicate the intervening sequence is palindromic. Long read sequencing is low-depth, and in light of the several-fold fewer assembled transcripts using Pychopper in the workflow (new Figs 3E, S3F, S3I, S3J), we would argue that Pychopper's discarding of useable reads (up to 8% of a dataset) may not be the optimal solution.

We are optimistic that the new direct comparisons of Pychopper to SLURP suggested by the reviewer can convince them that SLURP is not merely redundant but in fact, superior in terms of sensitivity, precision, and computational overhead (new Figs 3E, 3F, S3E, F, G, H, I, J).

On the positive side, the paper contains some informative analyses of lncRNA and cheRNA. Overall, the paper reads more like a biological analysis and would (after addressing below major comments) fit better in such a venue than a Computational Biology venue.

We thank the reviewer for noting the potential biological impact of this work. However, we contend that this paper is appropriate for *PLoS Computational Biology* readership, for the following reasons:

i) Systematic development of a new computationally inexpensive pre-processing method (SLURP): We report on the development of a stranding pipeline that outcompetes the existing methods for the assembly of the highest number of correct transcripts for spike-in standards, and results in the assembly of far more human and mouse transcripts with better precision. The development involved careful examination of the prevalence and location of sequencing primers, deciphering the most effective combinations of primers, using their locations to minimize non-specific hits, optimizing the number of mismatches and rigorous benchmarking against the contemporary methods. Its superior performance against UNAGI, Pychopper and ReorientExpress indicates that SLURP is likely to be a useful tool for future studies by others dealing with similar libraries.

ii) Discovery of an artifact that impacts transcript assembly: We report on the widespread occurrence of a cDNA synthesis artifact that negatively impacts transcript assembly as well quantitation. While we agree with the reviewer that Pychopper‘s handling of reads with artifactual primer configurations by discard does correct for this artifact, it does not report on the features of the intervening sequence nor account for the impact on alignment, assembly and quantitation that this discard imposes. Our detailed analyses show that these sequences are palindromic in nature and their dual alignment if retained impacts transcript assembly as well as quantitation, as does their discard. This can have a detrimental effect on differential gene expression analyses central to many transcriptomic studies.

iii) Development and benchmarking of a novel transcript assembly method: We provide analyses of the strengths and weaknesses of short- and long-read transcript assembly. Then, taking cues from those investigations and incorporating our stranding pipeline, we developed TASSEL to merge short- and long-read transcriptomes. Our thorough benchmarking against the current gold standard methodologies (including the host of those helpfully suggested by the reviewer) demonstrate that TASSEL shows the highest sensitivity of transcript assembly with equivalent or better precision. Going further than transcript assembly, we report that TASSEL captures some of the most important molecular features of a transcript such as its transcription start and end sites using orthogonal data such as H3K4me3 occupancy, Pol II ChIP, PRO-seq and highly curated datasets of transcript ends. As exemplified by our cheRNA analyses, we believe that TASSEL’s increased sensitivity

of transcript assembly, **especially for unknown transcripts**, will enable future studies to look deeper into the transcriptomes.

We hope that our original and new analyses convince the reviewer that our work will prove useful to *PLoS Computational Biology* readers performing similar analyses as well as transcriptomic studies focused on the identification and quantitation of complex transcripts.

Major

R4.1 L76-96. The authors discuss state-of-the-art tools but misses some of the work that is related most closely related to this paper. For the error-correction discussion, they miss TALC [1] (a hybrid tool). Other non-hybrid long-read approaches exist for ONT transcript assembly [2-7]. Long cDNA reorientation and chimeric removing tools exist: lima (PacBio), pychopper (ONT), and academic tools: Reorientexpress [8], mandalorian pipeline [9].

We thank the reviewer for suggesting other pipelines to compare with SLURP and TASSEL. In the revised manuscript, we provide a detailed comparison of SLURP and Pychopper. We report that SLURP *i)* strands significantly a greater number of reads than Pychopper (new Fig S3A) *ii)* shows higher sensitivity of transcript detection with equivalent or higher precision than Pychopper (new Figs 3E, S3E, F, G, H, I, J), and *iii)* has markedly lower computational load than Pychopper for a given set of reads (new Fig 3F, S3I and S3J).

As suggested by the reviewer, we also tested Reorientexpress (RExpress). However, we observed that RExpress failed to process many of the larger datasets (for example, dataset from the SG-Nex consortium with ~7-8 million reads per replicate) due to its higher memory requirement (>220 Gb RAM). We note that in the original paper, RExpress was tested either on a low-depth human cDNA sample (962,598 reads) or small transcriptomes of model organisms such as *S. cerevisiae* and *C. albicans*. The prohibitively high memory requirement of RExpress is perhaps also reflected in its lack of application in the literature, save the original paper. Even for the samples in which we were able to run RExpress, we report that it performs worse than Pychopper and SLURP (Response Figure 1). Due to its failure to process many of the datasets tested in our study and poor performance relative to SLURP and Pychopper, we reason that RExpress is not as good a candidate as Pychopper to benchmark SLURP. Rather further bloating an already dense manuscript's readability, we present this comparison in the response letter (Response Figure 1). In the event of publication in this journal, the responses will be published along with the paper so the true aficionados will have access to all these analyses.

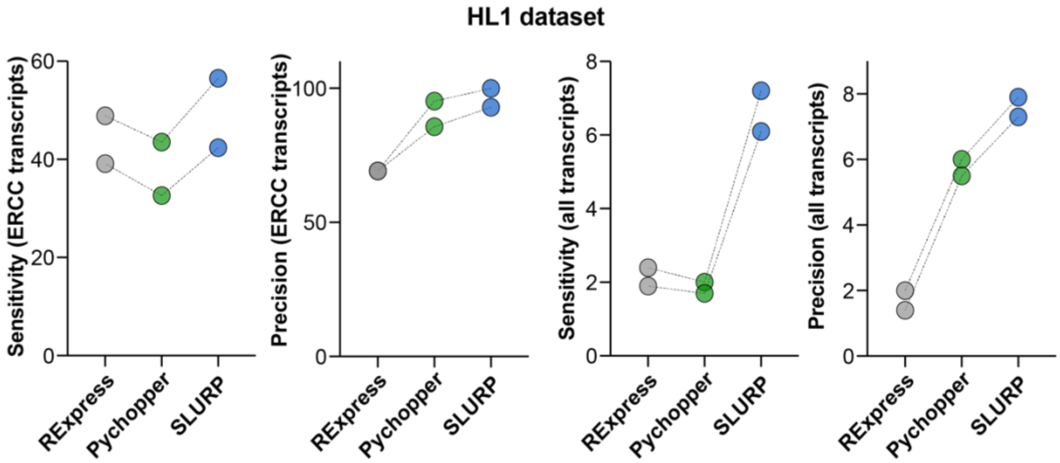

Response Figure 1. Comparison of ReorientExpress (RExpress), Pychopper and SLURP for sensitivity and precision of ERCC or all transcripts in the HL1 dataset. For RExpress, reads in the fastq files were processed using the mouse MLP models from the authors using `reorientexpress.py -predict -data input.fastq -model Mm_transcriptome_mlp.model -source experimental -output rexpress_output`. Processed reads from RExpress, Pychopper, and SLURP were then aligned using minimap2 followed by transcript assembly using StringTie2 in the long-read mode (`-L`).

As per the reviewer's suggestion, we investigated the Mandalorion pipeline originally used for assembling single-cell long reads. We note that Mandalorion is specifically designed for either ONT reads prepared by the Rolling Circle Amplification to Concatemeric Consensus (R2C2) method or PacBio CCS data. As noted by the authors of Mandalorion, it may not be suitable for regular ONT reads (https://github.com/christopher-vollmers/Mandalorion) (Response Figure 2).

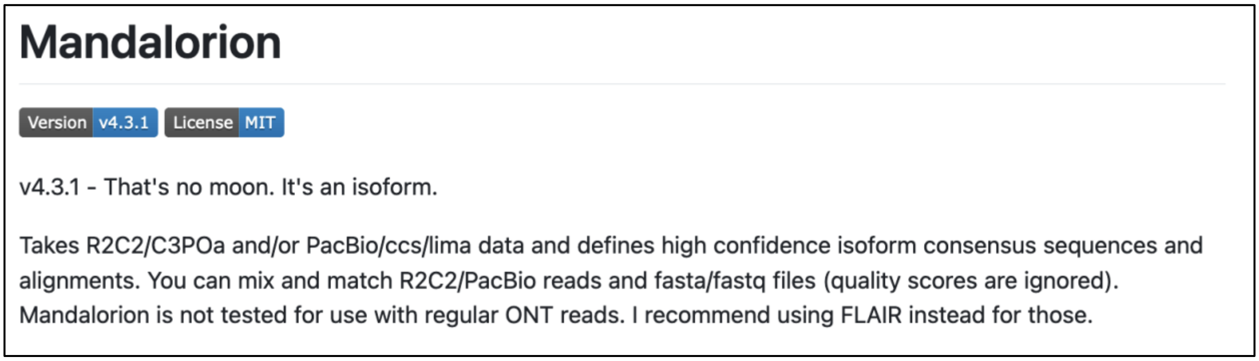

Response Figure 2. Snapshot from the Mandalorion github repository suggesting that Mandalorion is more suitable for R2C2- or PacBio-derived reads but not for "regular ONT reads".

Nonetheless, we tested Mandalorion and observed that TASSEL performs vastly better than Mandalorion for both the sensitivity and precision of transcript assembly (Response Figure 3). As Mandalorion is not designed to be used for typical ONT reads, we reason that it is not fair to compare it with general purpose assembly pipelines such as StringTie Mix, Bambu, FLAIR, and TASSEL. For similar reasons to RExpress, we think this is more germane to a response figure than a manuscript figure.

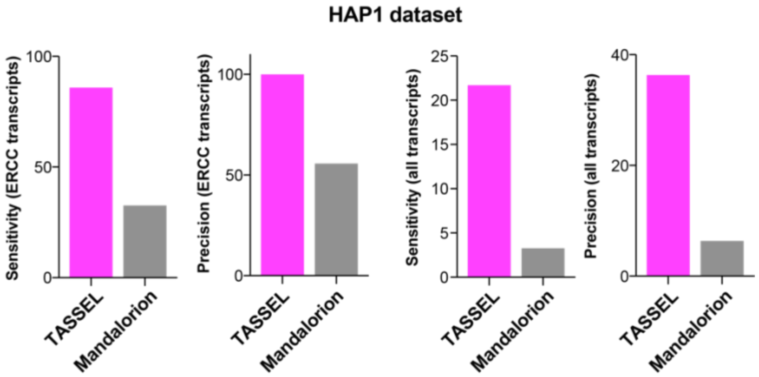

Response Figure 3. Comparison of TASSEL and Mandalorion for sensitivity and precision of ERCC or all transcripts in the HAP1 datasets. For Mandalorion, reads were pre-processed using the default settings (`./removePolyA.py -i input_fastq.fq -o processed_reads.fq -t n1, n2`). Processed reads were then subjected to the Mandalorion pipeline (`./Mando.py -p ./mando_output/ -g reference_annotation.gtf -G reference_fasta.fa -f processed_reads.fq`)

Next, we tested TAMA. We found that TAMA is highly reliant on reference annotation as it was able to assemble only one matching sequin transcript (compared to 107 for TASSEL) in the absence of reference annotation at the merge stage (new Fig S5C). We further probed this effect by providing 50% sequin annotations (and "hiding" the other 50%) at the merge stage and found that it assembled only one matching "hidden" sequin transcript (compared to 48 for TASSEL) (new Fig S5C). We speculate that when "TAMA_merge" is done on the assembled sample transcript annotation files by including a reference annotation file, TAMA includes transcript models from the reference annotation to its output often irrespective of whether they are present in the sample. This not only limits its capacity to assemble accurate *de novo* transcripts but also artifactually inflates its performance for transcripts it failed to assemble in the first place. Therefore, TASSEL performs better than TAMA for the assembly of known as well as unknown transcripts.

As suggested by the reviewer, we also compared TASSEL to non-hybrid methods: RNA-Bloom2, IsONform, IsoQuant, and RATTLE. We find that TASSEL shows the highest sensitivity and precision of transcript assembly for spike-in gold standards (new Figs 5B and 5E).

Finally, we sought to test TALC. However, we encountered several challenges to install and run TALC. We reached out to the authors for possible solution but unfortunately did not receive any communication from them (in two weeks). However, Shumate et al., (*PLoS Comp Bio*, 2022) tested StringTie Mix against TALC where authors reported that StringTie Mix performs better than TALC (From Shumate et al., *PLoS Comp Bio*, 2022*: "TALC is far slower than StringTie (running on a single RNA-seq samples takes TALC a day or longer, compared to less than one hour for StringTie), and it does not improve transcript assembly as compared to our new hybrid assembly algorithm."*). As our

detailed analyses (Figs 5B-J and S5A-F) show that TASSEL outperforms StringTie Mix, we reason that it is fair to surmise that TASSEL would also perform better than TALC.

We acknowledge that many pipelines exist for long-read transcript assembly but our original and new comparisons against the current gold standards demonstrate that TASSEL could be a valuable resource for a wide range of transcriptomics.

R4.2 L313-316 and the following section: It seems the authors did not use pychopper (https://github.com/epi2me-labs/pychopper) to identify, orient and trim full-length reads before alignment, as is standard (or any of the already existing academic alternatives such as Reorientexpress [8], mandalorian pipeline [9]). Consequently, at best, SLURP perhaps improves over pychopper or other computational read-orienters but is not benchmarked. At worst, SLURP may be redundant.

We thank the reviewer for raising this important point to benchmark SLURP against other stranding pipelines such as Pychopper, ReorientExpress and Mandalorion. As detailed above in point R4.1, we have now benchmarked SLURP against Pychopper and report that SLURP does improve over Pychopper in terms of sensitivity, precision and computational overhead (new Figs 3E-F, S3E-J). Similarly, SLURP performs vastly better than ReorientExpress (Response Figure 1).

R4.3 Section starting at L377: "these analyses reveal a widespread heretofore unknown artifact" Identifying chimeric transcripts due to e.g. RT-PCR artifacts is standard in long-read primer trimmers such as pychopper for ONT and lima (https://lima.how/) for PacBio and such artefacts are well known. The authors should look up "full-length non-chimeric reads" (see eg https://www.ncbi.nlm.nih.gov/pmc/articles/PMC6590394/) which was a standard concept at least a few years ago. Most analysis tools are designed to process full-length non-chimeric reads after pychopper/lima has been applied.

We agree that that many other artifacts are reported for long-read sequencing. However, as far as we know, this particular palindromic artifact is not noted in the literature. For ONT reads, we agree that Pychopper indicates the presence of artifactual occurrence of primers but in no way informs on the palindromic nature of the intervening sequences. Apart from the Pychopper's "warning", we could not find any report of the prevalence, origin, or downstream effects of such an artifact. Taking the reviewer's suggestion into account, we have now acknowledged Pychopper's "warning" about the presence of artifactual primer configurations in the revised manuscript (see lines 402-405).

Lima, as the reviewer alludes to, is designed for PacBio reads. The PacBio platform generates closed circular sequences (CCS) where the circular hairpin-ligated "dumbbell" cDNA molecule is sequenced by SMRT sequencing leading to concatenated palindromic reads. The lima package then de-concatenates these CCS to generate full

length non-chimeric/concatemer (FLNC) reads (https://isoseq.how/clustering/high-level-workflow.html). The generation of CCS and their processing by lima is very much an inherent part of the Iso-seq pipeline for the PacBio platform where the palindrome is deliberately generated by library preparation **after** reverse-transcription. The paper cited by the reviewer also describes SMRT sequencing used by the PacBio platform. We contend that the intentional elements of library preparation which result in palindromes post double-stranded cDNA library preparation, are very different from the artifact that we are reporting here for the ONT platform, which occurs during the RT-step. We note that our prior submission did include lima as does our current version (see lines 700-704), specifically showing that even after pre-processing by lima, transcript assembly from PacBio reads can benefit from TASSEL-based merge of short and long-read sequencing (Fig S5L).

As Pychopper does not inform on the full nature of the artifact, there is no prior literature describing this RT-artifact that we could find after diligent searching, and lima handles a different class of technology-specific chimeric reads, we feel it is reasonable to retain our description of this artifact in the manuscript.

R4.4 As a consequence of points 2 and 3, sections starting at L577 and L601 are not novel findings. Pychopper should be used, which should fix both these issues.
As detailed above, our original and new benchmarking analyses (upon the reviewer's suggestions) support the novelty of our work. In brief, *i)* the development and benchmarking of a computationally inexpensive stranding pipeline, SLURP, that outcompetes existing methods. We are thankful to the reviewer for suggesting Pychopper, as our new comparison of SLURP and Pychopper directly makes the case for the value of SLURP for future studies; *ii)* the detailed investigation of the prevalence and consequence of a wide-spread artifact that can negatively impact transcriptomic studies and, most importantly, *iii)* development of a new hybrid transcript assembly method, TASSEL, benchmarked against contemporary standalone and hybrid assembly methods on a diverse set of data. TASSEL outperformed all the pipelines (including the ones suggested by the reviewer) that we tested here. In light of these revisions, we hope that the reviewer agrees that our work represents a meaningful technical advance for the field with some novel aspects.

Minor
- L126: clarify which types "other long-read datasets" refers to.

We have now refined the statement to reflect the use of SLURP on ONT-derived cDNA reads and use of TASSEL on both ONT and PacBio reads (see lines 126-129).

- L156: Missing reference to tool used for short read alignments.

We have now included the reference to hisat2 used for short-read alignment (see line 158).

- L321-L322: "inaccurate alignment of reads to the incorrect strand" - it is not the alignment that is inaccurate.

We have now corrected the statement to “alignment of reads to the incorrect strand” (see lines 322-323).

- L562: "we systematically compared multiple transcript assembly approaches". This is an overstatement, as many state-of-the-art methods are missing.

We acknowledge that many pipelines exist for long-read RNA-seq. In the revised manuscript, taking the reviewer’s suggestions into account, we have now compared TASSEL with other stand-alone (Bambu, FLAIR, isONform, IsoQuant, Mandalorion, RATTLE, RNA-bloom2, StringTie2, TALON and TAMA) and hybrid (FLAIR with short-read correction, StringTie Mix, RNA-Bloom2 with short-read correction) pipelines on the same set of data. We think that that our detailed investigations do merit the use of the description: "we systematically compared multiple transcript assembly approaches." Notably, we claim to test “multiple” and not “all” approaches. Given the profusion of new computational methods in this space, it is impossible to be exhaustive, but thanks to the further suggestions of this reviewer, we feel that we have now more systematically compared all the reasonably similar/competitive tools that we are aware of.
